# Supplementary material for: GPR101 drives growth hormone hypersecretion and gigantism in mice via constitutive activation of Gs and Gq/11
Source: Nat Commun. 2020 Sep 21;11:4752. doi: 10.1038/s41467-020-18500-x (PMC7506554; doi:10.1038/s41467-020-18500-x)
Supplement: Supplementary file 1 — Supplementary Information [file 41467_2020_18500_MOESM1_ESM.pdf]

GPR101 drives growth hormone hypersecretion and gigantism via constitutive activation of G_s and $G_{q/11}$

Supplementary information

Abboud *et al.*

Supplementary Figures 1-5

Supplementary Tables 1-2

Supplementary Methods

Supplementary Figures

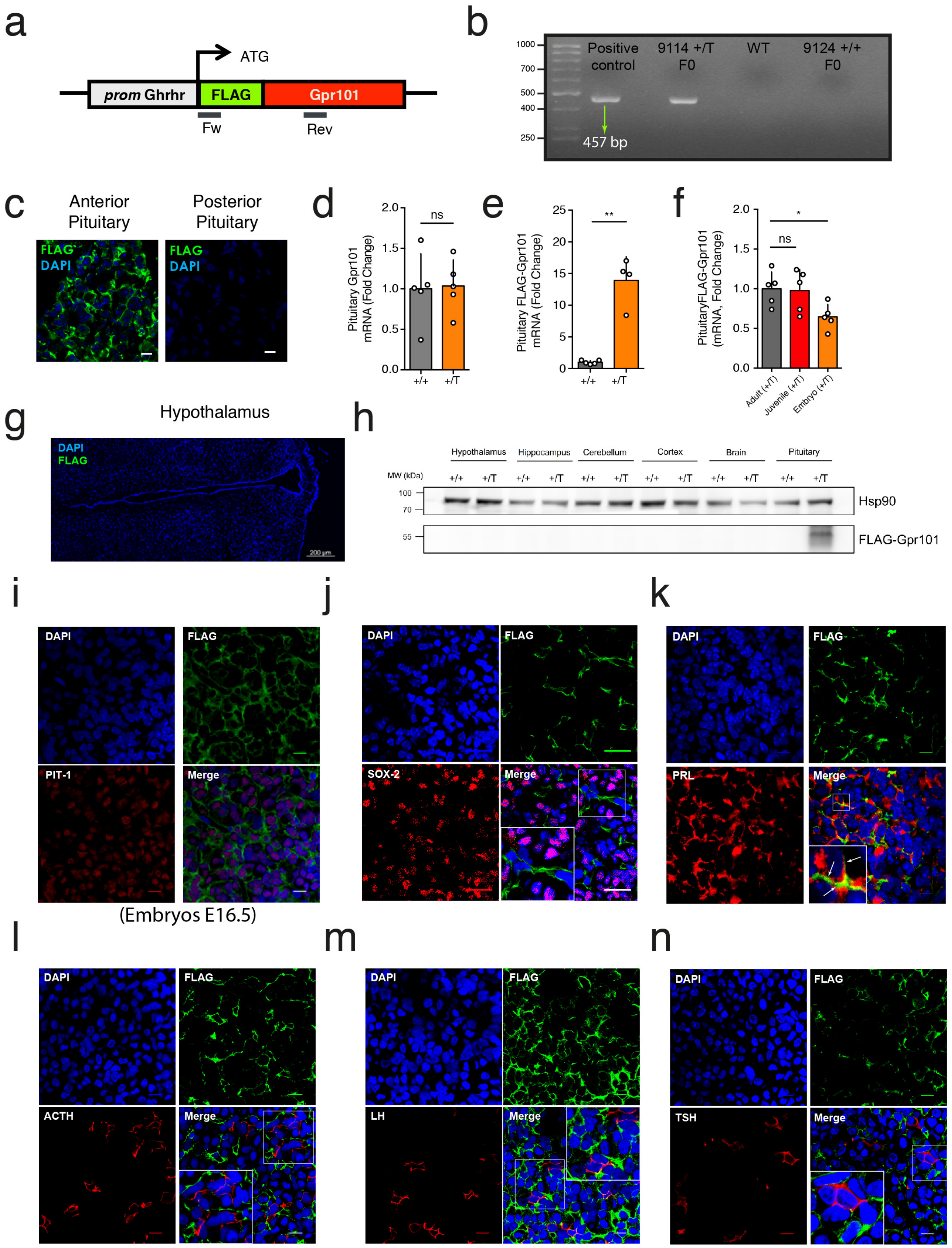

Supplementary Figure 1. Validation of functional expression of Gpr101 in a transgenic mouse model. a. DNA construct used to generate the *Ghrhr*^{Gpr101} mouse model. **b.** Genotyping of WT (+/+) and *Ghrhr*^{Gpr101} (+/T) mice. F0 founder mice were screened for FLAG-Gpr101 transgene inclusion. Forward and reverse primers (indicated in panel **a**) were designed to target the specific FLAG epitope and the Gpr101 sequence, respectively. A band is detected at 457bp. The plasmid containing the transgene FLAG-Gpr101 was used as a positive control, while the WT sample was considered as the negative control. This experiment was repeated at least 4 times. **c.** Immunofluorescent staining of anterior and posterior pituitaries from 29 week-old *Ghrhr*^{Gpr101} mice. FLAG-Gpr101 was labeled with antibodies directed against the FLAG epitope (green) indicating that this receptor is expressed in the anterior pituitary (and not in the posterior pituitary) of transgenic mice. DAPI is in blue. (*x*60 magnification, scale bar: 10µM). This experiment was repeated at least 3 times. **d.** The expression of native Gpr101 in the pituitary of WT (+/+) and *Ghrhr*^{Gpr101} (+/T) mice was quantified by RT-qPCR. Gapdh was used as a housekeeping gene control. n=5 independent samples are shown by genotype (mice aged 27 weeks, p>0.9999). **e.** The expression of FLAG-Gpr101 transgene in the pituitary of WT (+/+) and *Ghrhr*^{Gpr101} (+/T) mice was quantified by RT-qPCR. Gapdh was used as a housekeeping gene control. n=5 independent samples are shown by genotype (mice aged 27 weeks, p=0.0079). **f** Comparison of mRNA copies of FLAG-Gpr101 at different development stages (embryonic, juvenile [3 to 8 weeks] and adult) of *Ghrhr*^{Gpr101} (+/T) mice. n=5 mice per group, p>0.9999 for juvenile (+/T) *vs* adult (+/T) mice and p=0.0397 for (+/T) embryos vs (+/+) adult mice. **g.** Immunofluorescent staining of the hypothalamus of 29 weeks-old *Ghrhr*^{Gpr101} with the anti-FLAG antibody (Green). This experiment was repeated at least 3 times. **h.** Western Blot analysis of brain extract lysate from 29 weeks-old mice from both genotypes (+/+ and +/T mice). Membranes were incubated with either anti-FLAG or anti-Hsp90 antibody used as a loading control. The FLAG-Gpr101 band was detected at 57 kDa. Samples were run on the same gel and were cropped only for the purpose of this figure. Full scans of blots are available in the Source Data file. This experiment was repeated 3 times. **i-n.** Immunofluorescent staining of anterior pituitaries from 29 week-old *Ghrhr*^{Gpr101}. FLAG-Gpr101 appears in green and was double stained with antibodies against (in red) (**i**) Pit-1 antibody in E16.5 embryos, (**j**) the progenitor marker Sox2, (**k**) Prolactin (PRL), (**l**) Adrenocorticotropic hormone (ACTH), (**m**) Luteinizing hormone (LH), and (**n**) Thyroid-stimulating hormone (TSH). The nuclear DAPI staining is in blue. (*x*60 magnification, scale bar: 10µm). The staining experiments **(i-n)** were repeated at least 3 times. All data are Mean ± S.D. of at least 4 independent experiments. For statistical analysis of all data, a two-sided Mann-Whitney test was used. ns: not significantly different; * p<0.05; ** p<0.01; *** p<0.001. Source data are provided as a Source Data file.

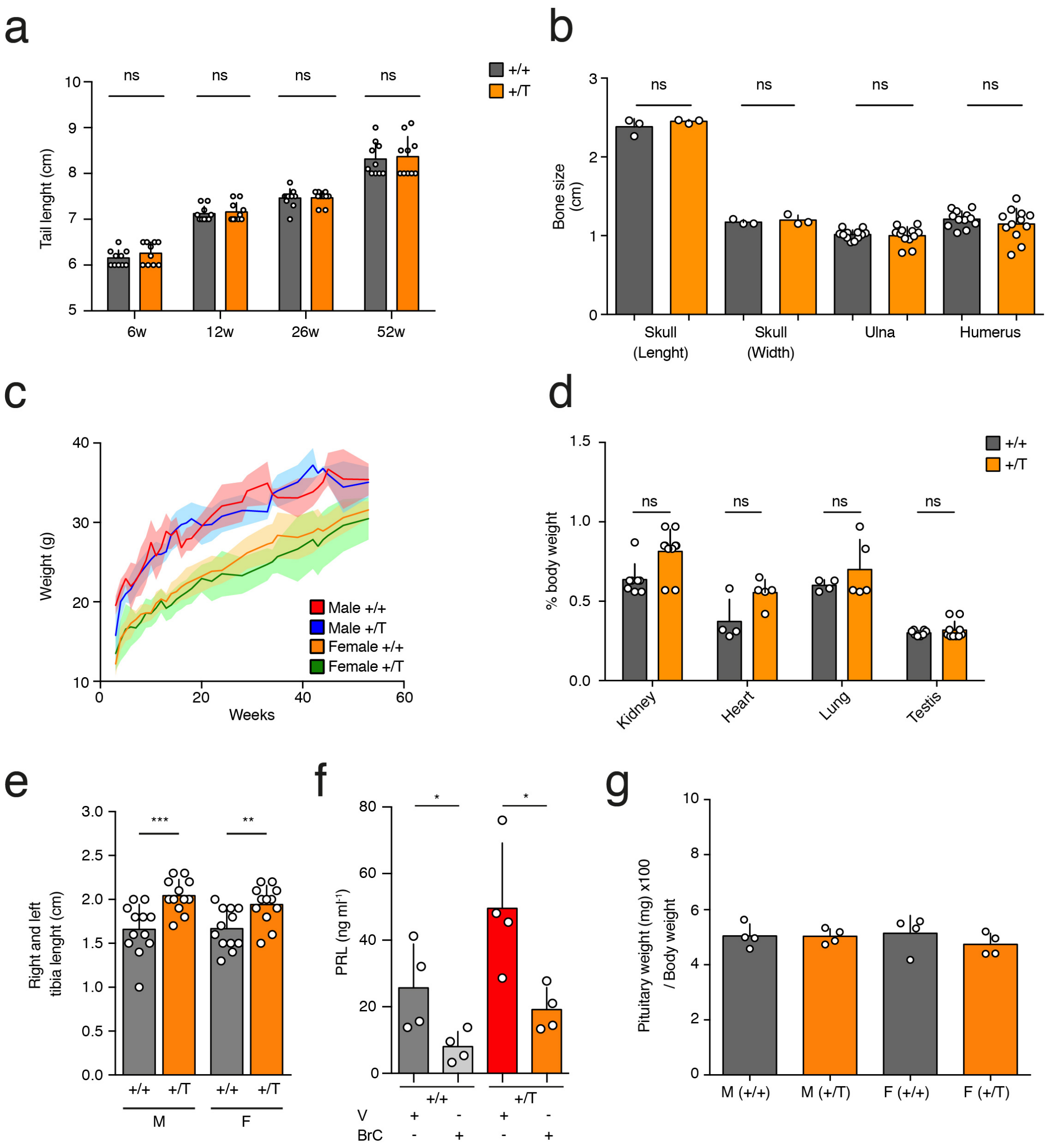

Supplementary Figure 2. Impact on GH, metabolism and bones. a. Tail length at several ages (6, 12, 26 and 52 weeks) for both genotypes (n=10 mice per group, males and females). **b.** Bone parameters such as the skull length (n=3 mice per group) and width (n=3 mice per group), the ulna (n=12 right and left ulna of 6 mice per group) and the humerus (n=12 right and left humeri of 6 mice per group) of WT (+/+) and *Ghrhr^{Gpr101}* (+/T) mice were measured by CT-scan and PMOD software. Statistical significance has been evaluated with two-sided Student t-test. **c.** Weight from 3 to 53 weeks of age for male (3-18 mice per group) and female (3-18 mice per group) mice of both (+/+) and (+/T) genotypes, respectively. **d.** The relative body weight percentage of organs (kidney, heart, lung and testis) was measured for WT (+/+) and *Ghrhr^{Gpr101}* (+/T) mice. For WT (+/+), n=4 mice (4 hearts, 4 lungs, 8 right and left testis, and 8 right and left kidneys) were used. For *Ghrhr^{Gpr101}* (+/T), n=5 mice (5 hearts, 5 lungs, 10 right and left testis, and 10 right and left kidneys) were used. **e.** Measurement of right and left tibial length for male and female mice from both genotypes (n=12 tibias from 6 mice per group, Males: p=0.0007 and females: p=0.0063). **f.** Evaluation of primary pituitary cells response to dopamine receptor agonists. Cells obtained from WT (+/+) or *Ghrhr^{Gpr101}* (+/T) animals were stimulated by Bromocriptine (BrC, 100nM), a D_2 receptor agonists or Vehicle control (n=4 independent samples, p=0.0286 for BrC-treated (+/+) vs vehicle-treated (+/+) pituitaries and p=0.0286 for BrC-treated (+/T) vs vehicle-treated (+/T) pituitaries. **g.** Weight, normalized by body weight, of the pituitaries isolated from males and females of both genotypes (n=4 mice per group). All data are Mean ± S.D. For statistical analysis of all data, a two-sided Mann-Whitney test was used unless otherwise stated. ns: not significantly different; * p<0.05; ** p<0.01; *** p<0.001. M: Males; F: Females. W: Week. Source data are provided as a Source Data file.

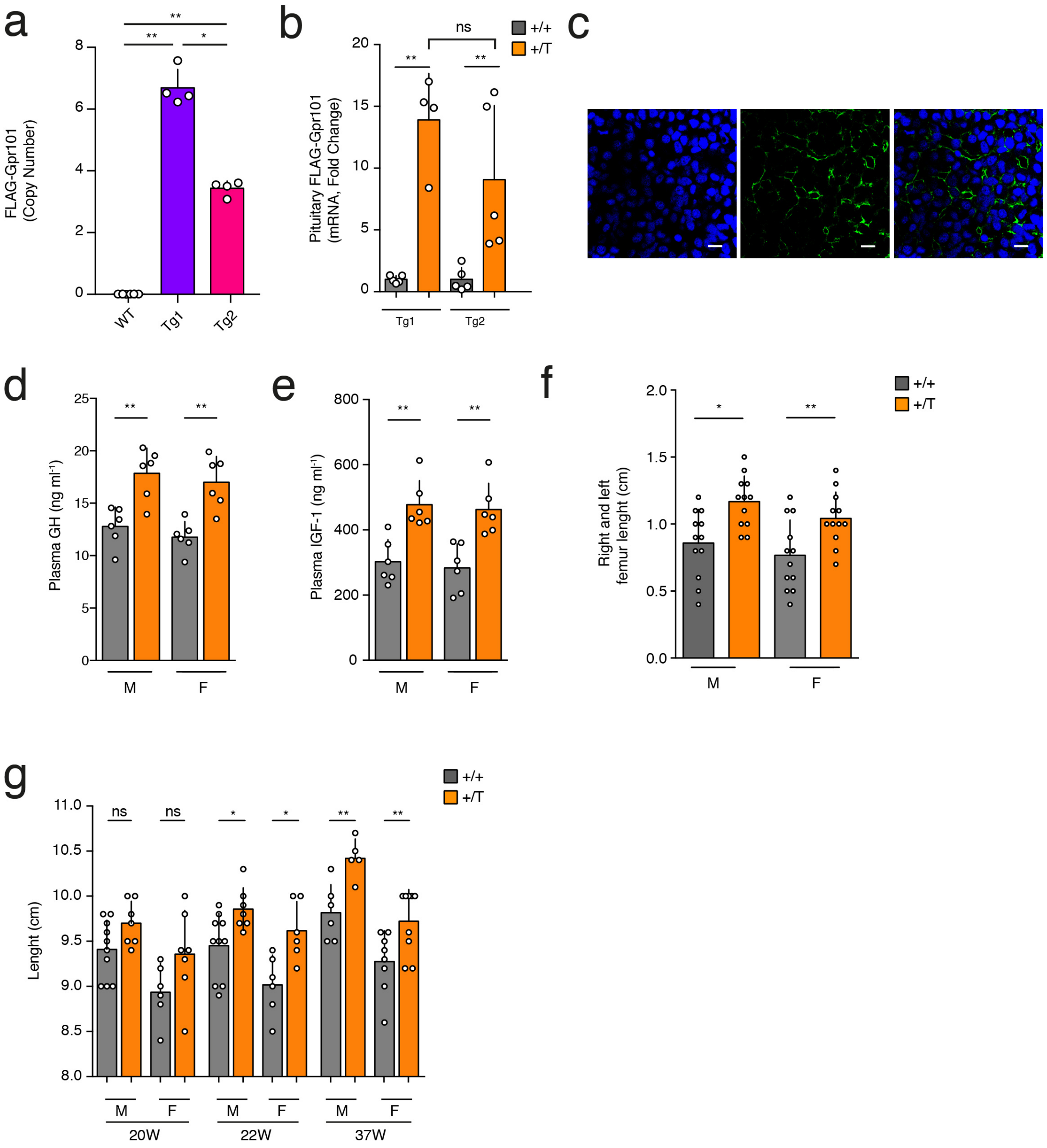

Supplementary Figure 3. Phenotypic analysis of another transgenic line. a. Determination of the transgene copy number in the genome of the different transgenic (Tg) *Ghrhr*^{Gpr101} lines (Tg1 and Tg2). The calculation was made with Gapdh normalization (n=8 WT mice and n=4 mice for each transgenic line, *p*=0.0020 for the comparison of the transgenic (Tg1 or Tg2) vs WT controls). **b.** Determination of transgene mRNA by RT-qPCR in both transgenic lines (n=5 mice per group, *p*=0.0079 for the comparison of each transgenic (Tg1 or Tg2) *vs* its control (WT1 or WT2), respectively. *p*=0.1508 for Tg2 vs Tg1). **c.** Representative Immunofluorescent staining of the anterior pituitary of the transgenic line 2 (Tg2) with anti-FLAG antibody. The nuclear DAPI staining is in blue. (*x*60 magnification, scale bar: 10µm). This experiment was repeated at least 3 times. **d.** Determination of plasma levels of GH in WT (+/+) and *Ghrhr*^{Gpr101}*_Tg2* (+/T) mice (n=6 mice per group, males: *p*=0.0087 and females *p*=0.0043). **e.** Determination of plasma levels of IGF-1 in WT (+/+) and *Ghrhr*^{Gpr101}*_Tg2* mice (n=6 mice per group, *p*=0.0022 for males and females. **f.** Quantification of femur length (n=12 right and left femurs from 6 mice per group, males: *p*=0.0390 and females: *p*=0.0087. **g.** Quantification of body length (nose-to-anus) at different age for mice of both genotypes from the transgenic line 2 (Tg2). The length of males was measured at the age of 20 (10-7 mice/group), 22 (10-7 mice/group), and 37 weeks (6-5 mice/group) for WT (+/+) and *Ghrhr*^{Gpr101} (+/T) mice, respectively. The length of females was measured at the age of 20 (6-7 mice/group), 22 (6-6 mice/group), and 37 weeks (8-9 mice/group) for WT (+/+) and *Ghrhr*^{Gpr101} (+/T) mice, respectively. Males: *p*=0.1006 (20W), *p*=0.0240 (22W) and *p*=0.0087 (37W). Females: *p*=0.0548 (20W), *p*=0.0108 (22W) and *p*=0.0022 (37W). All data are Mean ± S.D. For statistical analysis of all data, a two-sided Mann-Whitney test was used. ns: not significantly different; * p<0.05; ** p<0.01; *** p<0.001. F: Female; M: Male. W: week. Source data are provided as a Source Data file.

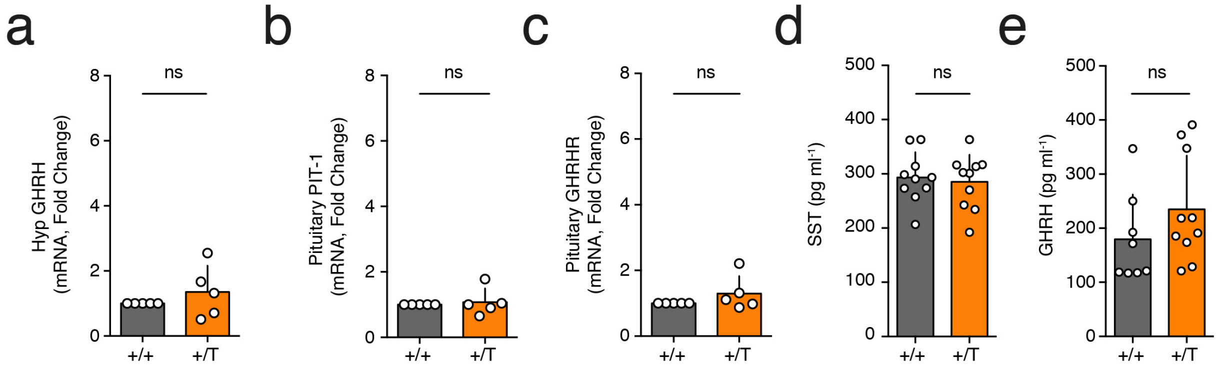

Supplementary Figure 4. Pituitary and hypothalamic hormone and receptor expression in mice. a-c. The expression of GHRH in the hypothalamus (**a**), as well as PIT-1 (**b**) and GHRHR (**c**) in the pituitary of WT (+/+) and *Ghrhr*^{Gpr101} (+/T) mice, was quantified by RT-qPCR. Gapdh was used as a housekeeping gene control. n=5 mice per group, aged at 27 weeks. For the hypothalamic GHRHR and pituitary PIT-1, p=0.1349. For the pituitary GHRHR, p=0.4206. **d&e**. Determination of plasma levels of Somatostatin (SST, **d**) and GHRH (**e**) in WT (+/+) and *Ghrhr*^{Gpr101} (+/T) mice (n=10 mice per group, mice aged 27 weeks). Statistical significance has been evaluated with two-sided Student t-test. SST: p=0.1501, GHRH: p=0.7161. All data are Mean ± S.D. For statistical analysis of all data, a two-sided Mann-Whitney test was used, unless otherwise stated. ns: not significantly different; * p<0.05; ** p<0.01; *** p<0.001. Source data are provided as a Source Data file.

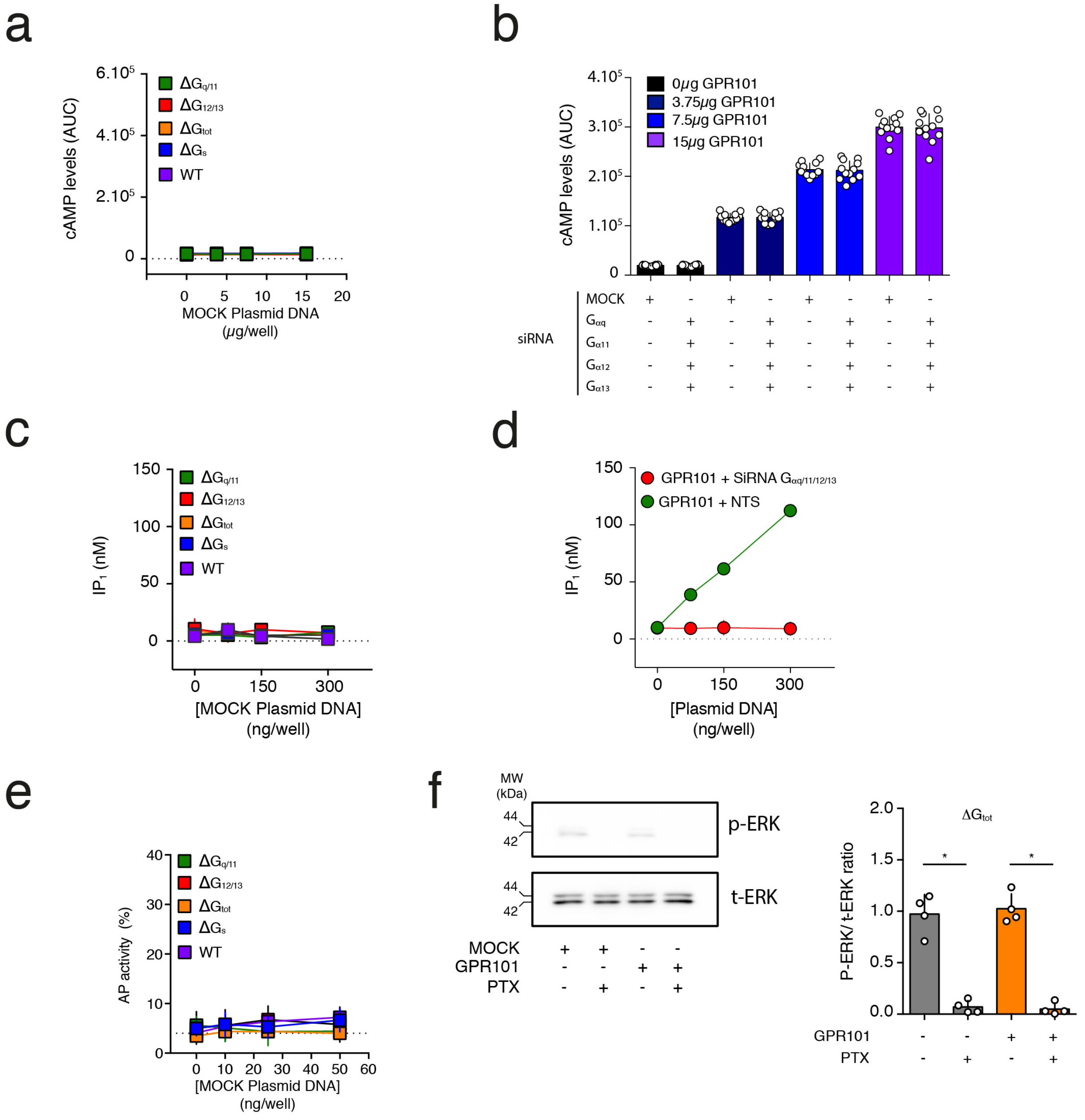

Supplementary Figure 5. Control experiments for GPR101-G protein coupling. a. cAMP measurement in HEK293 WT, $\Delta G_{\alpha s}$, $\Delta G_{\alpha q/11}$, $\Delta G_{\alpha 12/13}$ or ΔG_{tot} cells transfected with the Glosensor (15µg) and an empty vector (MOCK). **b.** cAMP measurements in pGlo.HEK293 cells treated with indicated siRNAs after the transfection of increasing concentrations of GPR101 (0, 3.75, 7.5 and 15µg, n=12 independent experiments per condition). **c.** Determination of IP_1 levels by ELISA on HEK293 transfected with an empty vector (MOCK). n=4 independent transfections per condition. **d.** Determination of IP_1 levels by ELISA on HEK293 treated with a combination of siRNA directed against $G_{\alpha q}$, $G_{\alpha 11}$, $G_{\alpha 12}$ and $G_{\alpha 13}$ and transiently transfected with GPR101-containing vectors. n=4 independent transfections per condition. **e.** TGF_{α} Shedding assay performed on HEK293 WT, $\Delta G_{\alpha s}$, $\Delta G_{\alpha q/11}$, $\Delta G_{\alpha 12/13}$ or ΔG_{tot} transiently transfected with empty vector (MOCK). Results are expressed as the percentage of AP activity in the conditioned medium. n=12 independent experiments. **f.** Western blot membrane probed with p-ERK and total ERK antibodies. Cell lysates were obtained from HEK.ΔG_{tot} cell lines transfected with GPR101 or empty vector (MOCK) before treatment with vehicle or pertussis toxin (PTX, 100ng ml^{-1}). Full scans of blots are available in the Source Data file. Bars represent quantification by densitometry of n=4 independent experiments. Statistical significance has been evaluated with two-sided Mann-Whitney test. p=0.0286 for PTX-treated $MOCK^{+}$ vs vehicle-treated $MOCK^{+}$ cells and for PTX-treated $GPR101^{+}$ vs vehicle-treated $GPR101^{+}$ cells. All data are Mean ± S.D. ns: not significantly different; * p<0.05; ** p<0.01; *** p<0.001. Source data are provided as a Source Data file.

Supplementary Table 1

Gene	Primer sequence (5'à 3')	GenBank accession n.	Product length (pb)
Mouse *Gapdh*	F: GAAGCCCATCACCATCTTCC<br>R: TGGCATGGACTGTGGTCATG	NM_001289726.1<br>[https://www.ncbi.nlm.nih.gov/nuccore/NM_001289726.1]	329
Mouse *Gh*	F : TGGACAGATCACTGCTTGGC<br>R : ATGTAGGCACGCTCGAACTC	NM_008117.3<br>[https://www.ncbi.nlm.nih.gov/nuccore/NM_008117.3]	206
Mouse *Ghrh*	F: GATGGCATCTACGTGTCGC<br>R: GGGTGCTCTTTGTGATCCTC	NM_010285.3<br>[https://www.ncbi.nlm.nih.gov/nuccore/NM_010285.3]	95
Mouse *Ghrhr*	F: ATCTCCATTGTAGCCCTCTGCG<br>R: TGACTTGGAAAGCCGCCAGTAC	NM_001003685.3<br>[https://www.ncbi.nlm.nih.gov/nuccore/NM_001003685.3]	576
Mouse *Pit-1*	F: TCTGTGCCTTCCTGTCATTATGG<br>R: CCTCCGTTTCCTCTTCCTTTCG	NM_001362468.3<br>[https://www.ncbi.nlm.nih.gov/nuccore/NM_001362468.3]	495
Rat ß-actin	F: CCCTCTGAACCCTAAGGCCAACCG<br>R: GTGGTGGTGAAGCTGTAGCCACGC	NM_031144.3<br>[https://www.ncbi.nlm.nih.gov/nuccore/NM_031144.3]	285
Rat *Gh*	F: CCTTGTCCAGTCTGTTTGCCAAT<br>R: CTGGGATGGTCTCTGAGAAGCAG	NM_001034848.2<br>[https://www.ncbi.nlm.nih.gov/nuccore/NM_001034848.2]	162

Gapdh: Glyceraldehyde 3-phosphate dehydrogenase; *Gh*: Growth Hormone; *Ghrh*: Growth Hormone-Releasing Hormone; *Ghrhr*: Growth Hormone-Releasing Hormone Receptor; *Pit-1*: Pituitary-specific positive transcription factor 1.

Supplementary Table 1. List of primers used for RT-PCR experiments.

Supplementary Table 2

Patient code	Sex	Age at diagnosis	Genotype	Tumor IHC	Tumor max diameter (mm)	Invasion (y/n)
ACROwt1	M	40	*AIP*, *GPR101* wt	GH	21	no
ACROwt2	F	62	*AIP*, *GPR101* wt	GH	9	no
ACROwt3	M	27	*AIP*, *GPR101* wt	GH	17	no
ACROwt4	F	38	*AIP*, *GPR101* wt	GH	11	no
ACROwt5	M	20	*AIP*, *GPR101* wt	GH	40	yes
ACROwt6	F	53	*AIP*, *GPR101* wt	GH	10	no
AIPmut1	F	17	*AIP* p.Q146S	GH/PRL	17	yes
AIPmut2	M	28	*AIP* p.R128H	GH	20	no
AIPmut3	M	24	*AIP* p.R271W	GH/PRL	25	yes
X-LAG1	F	2	dupGPR101	GH/PRL	15	no
X-LAG2	M	10	dupGPR101	GH/PRL	30	no
X-LAG3	F	5	dupGPR101	GH/PRL	20	no

Supplementary Table 2. List of profiling characteristics of patients with acromegaly/gigantism.

ACRO: Acromegaly; AIP: Aryl hydrocarbon receptor Interacting Protein; Dup: Duplication; GH: Growth Hormone; Mut: Mutation; PRL: Prolactin; X-LAG: X-Linked Acrogigantism; Wt: Wild-type.

Supplementary methods

RNA extraction and RT-PCR

GH3 cells ($2x10^5$ cells) were plated in a 35mm-dish and the transfection with plasmids (MOCK or GPR101) was carried out after 24h, followed by H89 (10µM) or Calphostin C (10µM) treatment after 48h as previously described earlier. The day after, the cells were lysed in RLT buffer and total RNA was extracted using the RNeasy Mini Kit (Qiagen, Cat. No 74104) according to the manufacturer protocol. For mouse tissues, pituitaries were collected from 27 week-old *Gpr101^{Ghrhr}* +/+ and +/T mouse lines 1 and 2. Total RNA was isolated using the NucleoSpin RNA XS kit (Machery-Nagel, Cat. No MN740902.50), according to the manufacturer protocol. The A260/280 ratio of the isolated RNA was 1.8-2.0. The mRNA was reverse transcribed into single-stranded cDNA by using 1µg of total RNA and the iScript™ cDNA synthesis Kit (BIO-RAD, California, USA). After conversion to cDNA, real-time PCR was performed with Takyon™ No ROX SYBR® 2X MasterMix dTTP blue (Eurogentec, Cat. No UF-NSMT-B0701) in the ABI 7900 Thermocycler (Applied Biosystems). PCR primers used for amplification of rat and mouse genes are detailed in Supplementary Table S1. The PCR primers were designed using http://www.ncbi.nlm.gov/tools/primer-blast. All experiments were performed in triplicate. Expression levels of mRNAs were calculated by the $2^{-\Delta\Delta Ct}$ method of relative quantification, and normalized to housekeeping genes rat beta-actin or mouse Gapdh.

Genotyping Strategy

Gpr101^{Ghrhr} (+/T) mice carrying the transgene were initially identified by PCR-based genotyping using genomic DNA samples prepared from tail tip biopsy, using the following primers: Forward primer, 5'-ATAAAGATGATGATGATAAACCACC-3' ; Reverse primer, 5'-CGTCGGTTGGTCATCTTG-3'. This primer pair was designed to amplify the sequence spanning from non-endogenous FLAG epitope the coding sequence of the mouse Gpr101. PCR

was performed with the following conditions: Initial denaturation (94°C for 30s) followed by 30 cycles of 94°C for 30s, 53°C for 60s and 68°C for 87s, and terminated by a final extension (68°C for 5 min). The PCR product band (457 bp) was detected after 2% agarose gel electrophoresis. WT (+/+) mouse genomic DNA and the plasmid containing the construct to generate the transgenic animals were used as a negative and a positive control, respectively.

Transgene copy number assessment

Genomic DNA was isolated from tail biopsies of WT and $Ghrhr^{Gpr101}$ mice, and the concentration of the DNA was measured using a nanodrop spectrophotometer. In order to examine the FLAG-Gpr101 copy number, a quantitative standard curve was generated by preparing a series of standard samples (containing 10^8 to 10^1 copies of FLAG-Gpr101 transgene,) obtained by mixing together the WT genomic DNA with the plasmid that was used to generate the transgenic mice (pcDNA3.1 *prom*Ghrhr-FLAG-Gpr101). The initial concentration of the plasmid DNA was 2861ng μl^{-1} and the total vector length was 8891 bp. The required copy number was calculated using the following equation:

Copy number=(amount of DNA(ng) x 6,022 x 10^{23})/(vector length (bp) x 1 x 10^9 x 650).

Primer sequences used for the FLAG-Gpr101 transgene were: 5’-ATGGATTATAAAGATGATGATG-3’ (forward) and 5’-AGGTGAAGTAGCTGAATCATGGG-3’ (reverse). Mouse Gapdh was used as a reference gene. KOD SYBR® qPCR Mix (TOYOBO, Osaka, Japan, Cat. No F1236K) was used for the real time amplification according to the manufacturer protocol. The final concentrations of forward and reverse primers were 0.4µM. 100ng of genomic DNA were used per reaction (20µl final volume). Cycling conditions used were as follows: denaturation at 98°C for 10s, annealing at 60°C for 10s, and elongation at 68°C for 94s (40 cycles). Assays were performed in triplicate using the ABI 7900 HT (Applied biosciences) detection system. A no template control was

performed for both FLAG-Gpr101 and Gapdh primers in each qPCR experiment to rule out contamination of the master mix with genomic DNA. Cycle threshold (Ct) values for FLAG-Gpr101 and Gapdh were determined for each sample. ΔCt values were calculated by subtracting the transgene Ct value for a particular sample from its corresponding reference gene Ct value ($\Delta Ct = Ct_{FLAG\text{-}Gpr101} - Ct_{Gapdh}$). The quantitative standard curve was drawn by plotting ΔCt (on the Y axis) against known copy number of each standard (on the X axis) using a logarithmic scale. The resulting equation $\log_{10}$ (copy number) = -3.2423 ΔCt +20.939 (R^2 = 0.9903) was used to estimate copy number of samples based on the ΔCt value. FLAG-Gpr101 copy number per diploid cell was calculated by dividing the number of FLAG-Gpr101 copies in the DNA sample by the number of cells from which the DNA was isolated. It has been reported that each diploid cell from a C57BL/6J mouse to yield 6pg DNA (Capparelli et al., 1997). Thus, 100ng DNA ≈ 1.67×10^4 diploid cells. Two different mouse lines were generated (from the same construct) and analyzed (shown to have comparable patterns of transgene expression) to rule out any insertion site or copy number-dependent effect or ectopic expression of the transgene.

Immunofluorescent staining of FLAG-Gpr101 and pituitary markers

Pituitaries from 29 week-old *Ghrhr*^{Gpr101} and WT mice were fixed overnight with 4% paraformaldehyde (PFA) at 4°C and paraffin-embedded. The fixed tissues were sectioned at 5µm and mounted on Superfrost® Microscope Slides (Thermo ScientificTM, Cat. No 12372098). After deparaffinization and antigen heat retrieval (using citrate buffer at pH 6), sections were washed with PBS, and permeabilized at room temperature for 10min with PBS containing 0.5% Triton X-100. After the wash, they were blocked in blocking buffer (PBS containing 5% FBS and 0.5% Triton X-100) for 60min and then incubated with either a primary antibody [polyclonal rabbit anti-Sox2 (abcam, Cat. No ab97959, dilution 1:50), monoclonal rabbit anti-PRL (abcam, Cat. No ab238938, dilution 1:1000), polyclonal rabbit anti-ACTH

(Bioss antibodies, Cat. No bs-0443R, dilution 1:50), polyclonal rabbit anti-LH (FabGennix, Cat. No LH-101AP, dilution 1:50) or polyclonal rabbit anti-TSH (Bioss antibodies, Cat. No bs-2676R, dilution 1:50) antibody] overnight at 4°C. Sections were subsequently washed three times in PBS containing 0.5% Triton X-100, prior to a 2h room temperature incubation with the secondary antibody [anti-rabbit IgG (H+L) F(ab')2 fragment Alexa Fluor 647 conjugate (Cell Signaling Technology, Cat. No 4414, dilution 1:1000)]. After, sections were washed three times in PBS containing 0.5% Triton X-100 and incubated with a monoclonal mouse anti-FLAG antibody (Sigma–Aldrich, Cat. No F3165, clone M2, dilution 1:1000) for 2h at room temperature. Sections were again washed three times in PBS containing 0.1% Triton X-100 and incubated with the secondary antibody [anti-mouse IgG Fab2 Alexa Fluor 488 conjugate antibody (Cell Signaling Technology, Cat. No 4408S, dilution 1:1000)] for 2h at room temperature. Sections were then washed three times in PBS containing 0.5% Triton X-100 and mounted using ProLong Gold Antifade Mountant containing DAPI (ThermoFisher Scientific, Cat. No P36931). Finally, stained pituitaries were visualized by confocal microscopy and image acquisition was performed on a NIKON A1R (Tokyo, Japan) confocal microscope (oil immersion objective, *x*60 magnification). The same procedure was applied to co-stain pituitaries of *Ghrhr*$^{\mathrm{Gpr101}}$ embryos (E16.5) with rabbit anti-Pit1 antibody (dilution 1:500) and mouse anti-FLAG antibody (1:1000).

Immunofluorescent staining of FLAG-Gpr101 in the brain

Ghrhr^{Gpr101} perfused brain tissue was collected and fixed in 4% PFA overnight at 4°C. Then, the tissue was incubated with 30% sucrose (for an additional day), embedded in OCT, and cryosectioned at 12µm. The brain section was dried (for 20min), and fixed in 4% PFA (for 10min), permeabilized in 0.1% Triton-X (for 10min), and blocked with 5%FBS (for 1h). Primary antibody for FLAG was added overnight (1:1000 dilution) and the anti-mouse secondary antibody coupled to AF488 (1 :1000 dilution) was applied for 2h at room

temperature. Slides were counterstained with DAPI and were mounted in Diamond Antifade Mountant. Reconstructed images of the third ventricle area from the arcuate nucleus of the hypothalamus were acquired by using the Zeiss confocal microscope (air dry objective, x20 magnification).

Western Blot for the detection of FLAG-Gpr101 in the brain

Several regions of the brain (pituitary, cortex, cerebellum, hippocampus and hypothalamus) of WT and *Ghrhr*^{Gpr101} mice (29-week-old) were homogenized in lysis buffer (PBS, 1%NP containing protease inhibitors) for 30min at 4°C, and debris were removed by centrifugation at 15000 rpm for 10min. Protein concentrations were determined by BCA protein assay. Equal amounts of proteins (40µg) were mixed with 1x Laemmli buffer and were incubated at room temperature for 1h. Proteins were separated on 4–10% SDS-PAGE gel and were transferred onto nitrocellulose membranes. Blocking was performed in 5% BSA. Membranes were incubated with primary antibodies for FLAG (mouse anti-FLAG antibody, Sigma–Aldrich, Cat. No F3165, clone M2, dilution 1:1000) and Hsp90 (rabbit anti-Hsp90 antibody, Cell Signaling Technology, Cat. No 4877T, dilution 1:1000) overnight at 4°C. Specie-specific HRP-conjugated secondary antibodies (anti-mouse IgG horseradish peroxidase (HRP)-linked antibody, Cell Signaling Technology, Cat. No 7076P2, dilution 1:1000) and anti-rabbit IgG horseradish peroxidase (HRP)-linked antibody (Cell Signaling Technology, Cat. No 7074S, 1:1000 dilution), respectively) were left on the membranes for 1h, and the signal was developed with ECL reagent and then imaged with the ImageQuant LAS 4000 detection system. Uncropped scans of the blots are supplied in the Source Data file.

Prolactin secretion in mouse pituitary primary cultures

Pituitaries from WT and *Ghrhr*^{Gpr101} mice (27 week-old) were collected and dissociated into cell suspensions by using the gentleMACS™ dissociator and the Adult Brain Dissociation Kit (Myltenyi, Cat. No 130-107-677). The cell suspensions were passed through the 70µm cell strainer, and were centrifuged at 300xg for 10min. The pellets were resuspended in DMEM culture medium supplemented with 10% FBS and 1% penicillin/streptomycin. After isolation, pituitary cells were directly seeded (at a cell density of $4x10^5$ cells per well) in a 12-well plate coated with poly-D-lysine, and maintained at 37°C in 5% CO_2 for 24h. The next day, cells were exposed to Bromocriptine (Tocris, Cat. No 0427, 100nM) or the vehicle (1% DMSO), and 48h later, culture media were collected and were stored at -80°C until further processing. Rat/Mouse Prolactin (Prl) ELISA Kit (ThermoFisher Scientific, Cat. No EMPRL) was used to assess the concentration of prolactin in the supernatant. The ELISA was performed according to the manufacturer instructions.

Determination of plasma hormones

The blood of 27 week-old *Ghrhr*^{Gpr101} and WT mice was collected from the inferior vena cava in EDTA capillary blood tubes (Greiner, Cat. No 450475), and the plasma was separated from cells by centrifugation (2000*xg)* for 10min. Plasma hormone levels were determined using commercial ELISA immunoassays according to the protocol of the manufacturers. Mouse somatostatin (SST) was detected and quantified by using the SST ELISA Kit (Mouse) (Biotechne, Cat. No ORB196567_96 assays). Mouse GHRH was detected and quantified by using the Mouse GHRH ELISA Kit (Sandwich ELISA) (Biotechne, Cat. No LS-F34871-1).
