# Supplementary material for: GPR101 drives growth hormone hypersecretion and gigantism in mice via constitutive activation of Gs and Gq/11
Source: Nat Commun. 2020 Sep 21;11:4752. doi: 10.1038/s41467-020-18500-x (PMC7506554; doi:10.1038/s41467-020-18500-x)
Supplement: Supplementary file 3 — Reporting Summary [file 41467_2020_18500_MOESM3_ESM.pdf]

nature research

Corresponding author(s): Julien Hanson
Albert Beckers

Last updated by author(s): Aug 5, 2020

Reporting Summary

Nature Research wishes to improve the reproducibility of the work that we publish. This form provides structure for consistency and transparency in reporting. For further information on Nature Research policies, see Authors & Referees and the Editorial Policy Checklist .

Statistics

For all statistical analyses, confirm that the following items are present in the figure legend, table legend, main text, or Methods section.

n/a	Confirmed	
☐	☒	The exact sample size (*n*) for each experimental group/condition, given as a discrete number and unit of measurement
☐	☒	A statement on whether measurements were taken from distinct samples or whether the same sample was measured repeatedly
☐	☒	The statistical test(s) used AND whether they are one- or two-sided<br>*Only common tests should be described solely by name; describe more complex techniques in the Methods section.*
☒	☐	A description of all covariates tested
☒	☐	A description of any assumptions or corrections, such as tests of normality and adjustment for multiple comparisons
☐	☒	A full description of the statistical parameters including central tendency (e.g. means) or other basic estimates (e.g. regression coefficient) AND variation (e.g. standard deviation) or associated estimates of uncertainty (e.g. confidence intervals)
☐	☒	For null hypothesis testing, the test statistic (e.g. *F*, *t*, *r*) with confidence intervals, effect sizes, degrees of freedom and *P* value noted<br>*Give P values as exact values whenever suitable.*
☒	☐	For Bayesian analysis, information on the choice of priors and Markov chain Monte Carlo settings
☒	☐	For hierarchical and complex designs, identification of the appropriate level for tests and full reporting of outcomes
☒	☐	Estimates of effect sizes (e.g. Cohen's *d*, Pearson's *r*), indicating how they were calculated

Our web collection on statistics for biologists contains articles on many of the points above.

Software and code

Policy information about availability of computer code

Data collection	CT-scan (eXplore micro-CT, Gamma Medica, GE Healthcare, USA): All micro-CT images were reconstructed using the Feld Kamp's filtered back projection algorithm with a cutoff at the Nyquist frequency to obtain a 3D volume with an isotropic voxel size of 100µm. The CT-scan data were collected with Host Console Interface and MicroView ABA 2.3.a7<br>Confocal microscopy: NIS-Element software from Nikon (Tokyo, Japan).
Data analysis	- PMOD 3.6 software (PMOD Technologies, Zurich, Switzerland; RRID:SCR_016547)<br>- Microsoft Excel (Microsoft Office, Microsoft®, USA, version 16.16.24 (200713)).<br>- GraphPad Prism v.6 (GraphPad Software, San Diego, CA, USA)<br>- ImageJ v.1.47 (National Institutes of Health, USA) bundled with Java 1.8.0_172.<br>- Adiposoft v1.15 plug-in software (Center for Applied Medical Research CIMA, University of Navarra, Spain).

For manuscripts utilizing custom algorithms or software that are central to the research but not yet described in published literature, software must be made available to editors/reviewers. We strongly encourage code deposition in a community repository (e.g. GitHub). See the Nature Research guidelines for submitting code & software for further information.

Data

Policy information about availability of data

All manuscripts must include a data availability statement. This statement should provide the following information, where applicable:
- Accession codes, unique identifiers, or web links for publicly available datasets
- A list of figures that have associated raw data
- A description of any restrictions on data availability

All the data and materials are available from the authors upon reasonable request. The source data underlying Figures 1A-1M, 2A–2I, 3A-3H, 4A-4I, 5A-5J, 6A-6G and 7A-7E as well as Supplementary Figures S1B-S1N, S2A-S2G, S3A-S3G, S4A-S4E and S5A-S5F are provided with this paper as a Source Data file. Uncropped and

unprocessed scans of blots can be found in the Source Data file provided with this paper.

Field-specific reporting

Please select the one below that is the best fit for your research. If you are not sure, read the appropriate sections before making your selection.

☒ Life sciences ☐ Behavioural & social sciences ☐ Ecological, evolutionary & environmental sciences

For a reference copy of the document with all sections, see nature.com/documents/nr-reporting-summary-flat.pdf

Life sciences study design

All studies must disclose on these points even when the disclosure is negative.

Sample size

When statistical tests were used, all experiments (including in vitro cell culture experiments), were carried out with at least n=4 independent samples/animal per group or n=4 individual experiments (except the skull measurements in Supplementary Figure 2b where n=3) in order to quantitatively compare samples between them.
For the measurement of hormones (GH, IGF-1, PRL) in mice, we determined our sample size according to existing literature. Several articles mentioned the use of n=4-6 animals per group to measure plasma hormones (n=4 for Sato et al., 2017 [Drug Metab Dispos. 2017 Jun;45 (6):586-592. PMID: 28330858], n=6 for Stewart et al., 2013 [Biochem J. 2013 Jan 15;449(2):401-13. PMID: 23075222]) in mice. The use of relatively small sample size is compatible with the large volume collected (approximately 500µl by inferior vena cava). Thus, the variability between samples is reduced, as compared with other blood collection methods such as tail tip (4µl) where sample size is typically higher (Stein et al. 2011 [Endocrinology. 2011 Aug;152(8):3165-71. PMID: 21586549]).

For the determination of GH in the supernatant of GH3 cells and transfection of SiRNA, we used n=6 independent transfections or samples. The size of our samples per group (n=6) is in similar range than other studies done on cells (n=4 wells/group for Kim et al., 2005 [Pituitary. 2005;8(2):155-62. PMID: 16379030]), n=4wells/group for Tani et al., 2015 [Sci Rep. 2015 Jun 3;5:10878. PMID: 26039928]).

For the measurement of body length, we used between 4-24 males and 8-13 females to quantify the body length for different groupes of age. For these kind of experiment, the constitution of larger groups (for example n=24 males and n=13 females per group) to give more robustness to our observations is compatible with animal experiment ethics because these experiments are non-invasive and with minimal stress, which permits repeated measures. Another important aspect that we took into account is the risk of loosing animal over a long period of time and the issue of having not enough data if the constituted group was too small.
We used n=6 mice per group to quantify bone length (right and left femurs and tibia). Other articles such as Stratikopoulos et al., 2008 [Proc Natl acad Sci USA. 2008 Dec 9;105(49):19378-83. PMID: 19033454] used approximately between 2 and 5 of animals per group.

For the glucose tolerance test, we used n=5 mice/group for males and n=6 mice/group for females. Other articles used similar number of animals per group (n=6 for Horakova et al., 2019 [Sci Rep. 2019 Apr 16;9(1):6156. PMID: 30992489], and n=3-11 animals per group for Dunn et al., 2009 [Endocrinology. 2009 Nov;150(11):4999-5009. PMID: 19819967]).

Data exclusions

No data were excluded

Replication

Each experiment has been repeated at least 3 times independently, unless stated otherwise. The precise number of replicates has been included in the legends. All attempts at replication were successful.

Randomization

Randomization was performed on cell cultures, mice groups and human biosamples. The cell cultures, mice and human biosamples were randomized prior to any measurements. For example, for experiment on cells, the different cell lines or transfection mix were distributed in microplate or other vessels with different patterns between experiments. Mice underwent the experimental procedure randomly without considerations of age, sex or genotype. Histological sections were distributed randomly and examined in various order under the microscope. For studies on immunohistochemistry in human pituitary adenomas, the tumor samples were allocated into groups according to previously determined genetic status: (a) X-LAG patients (n=3); (b) acromegaly patients without AIP mutations (n=6) and (c) acromegaly patients with AIP mutations (n=3). The allocation was controlled only to permit the inclusion of male and female subjects in each of the three groups; otherwise the samples were randomly chosen from available tumor tissues stock.

Blinding

Investigators were blinded for the measurements performed on mice and human bio samples. For mice, each subject was given a code number whose signification was know when the data were analysed. Human samples were processed and Stained/analysed by 2 different investigators who matched their records after the image acquisition. On cells-related experiments, the investigators were not blinded for practical reasons (usually only one investigator involved). However, the data collected were quantitative (measured by a plate reader for instance) and thus not subject to investigator bias.

Reporting for specific materials, systems and methods

We require information from authors about some types of materials, experimental systems and methods used in many studies. Here, indicate whether each material, system or method listed is relevant to your study. If you are not sure if a list item applies to your research, read the appropriate section before selecting a response.

Materials & experimental systems

n/a	Involved in the study
☐	☒ Antibodies
☐	☒ Eukaryotic cell lines
☒	☐ Palaeontology
☐	☒ Animals and other organisms
☐	☒ Human research participants
☒	☐ Clinical data

Methods

n/a	Involved in the study
☒	☐ ChIP-seq
☒	☐ Flow cytometry
☒	☐ MRI-based neuroimaging

Antibodies

Antibodies used

Cell Signaling Technology (Danvers, MA, USA)
- Anti-phospho-p44/42 MAPK (Erk1/2) (T202/Y204, D13.14.4E) rabbit monoclonal antibody (Cat. No 4370S)
- Anti-p44/42 MAPK (Erk1/2) rabbit monoclonal antibody (Cat. No 4695S)
- Secondary anti-rabbit IgG horseradish peroxidase (HRP)-linked antibody (Cat. No 7074S)
- Anti-FLAG mouse antibody (Cat. No 8146)
- Anti-HA rabbit antibody (Cat. No 3724)
- Anti-phospho-PKC-alpha/betaII (Thr638/641) rabbit monoclonal antibody (Cat. No 9375S)
- Anti-PKC-alpha rabbit monoclonal antibody (Cat. No 2056S)
- Anti-phospho-PKA(Thr197) mouse monoclonal antibody (Cat. No 4781S)
- Anti-PKA alpha rabbit polyclonal antibody (Cat. No 4782S)
- Secondary anti-mouse IgG horseradish peroxidase (HRP)-linked antibody (Cat. No 7076P2)
- Secondary anti-rabbit IgG Fab2 Alexa Fluor 647 antibody (Cat. No 4414S)
- Secondary anti-mouse IgG Fab2 Alexa Fluor 488 conjugate antibody (Cat. No 4408S)
- Anti-Hsp90 rabbit antibody (Cat. No 4877T)

LSBio (LifeSpan Biosciences, The Netherlands)
- Anti-GHRHR polyclonal rabbit antibody (Cat. No LS-B6566)

Novus Biologicals (Bio-Techne Ltd., United Kingdom)
- Anti-Pit1 polyclonal rabbit antibody (Cat. No NBP1-92273)

Sigma–Aldrich (St. Louis, MO, USA)
- Anti-FLAG mouse monoclonal antibody (Cat. No F3165, clone M2)
- Secondary anti-mouse-IgG-Atto 647N antibody (Cat. No 50185)

R&D Systems (Minneapolis, MN, USA)
- Anti-GH goat polyclonal antibody (Cat. No AF1067-SP)

Abcam (Cambridge, UK)
- Secondary anti-goat Alexa Fluor 488 conjugate antibody (Cat. No ab150129)
- Rabbit Specific HRP/DAB (ABC) Detection IHC kit (Cat. No ab64264)
- Anti-SOX2 polyclonal rabbit antibody (Cat. No ab97959)
- Anti-PRL monoclonal rabbit antibody (Cat. No ab238938)

Bioss antibodies (Bio-connect, Belgium)
- Anti-ACTH polyclonal rabbit antibody (Cat. No bs-0443R)
- Anti-TSH polyclonal rabbit antibody (Cat. No bs-2676R)

FabGennix (Kyser Way, TX, USA)
- Anti-LH polyclonal rabbit antibody (Cat. No LH-101AP)

Merck Millipore (Burlington, MA, USA)
- Anti-Ki-67 rabbit polyclonal antibody (Cat. No AB9260)

Life Technologies (Carlsbad, CA, USA)
- Anti-phospho-PKC alpha (Thr638) rabbit polyclonal antibody (Cat. No 44-962G)

Validation

The antibodies that were used have been either extensively described in the literature and/or validated by the provider. In addition, we validated ourselves the specificity of the AB by including positive controls in our experiments or by comparing tissues/cells with and without the antigen when possible.

A) All antibodies at CST are validated for immunofluorescence and/or western blot. The procedure at CST to perform the validation:
https://www.cellsignal.com/contents/resources/videos-and-webinars/CST-antibody-validation-documentary

Antibody validation at CST for immunohistochemistry. The procedure at CST to perform the validation:
https://www.cellsignal.com/contents/our-approach-antibody-validation-principles/antibody-validation-for-immunohistochemistry/ourapproach-validation-ihc

Antibody validation at CST for immunofluorescence:
https://www.cellsignal.com/contents/our-approach-antibody-validation-principles/antibody-validation-for-immunofluorescence/ourapproach-validation-if

Antibody validation at CST for western blot : The procedure at CST to perform the validation:
https://www.cellsignal.com/contents/our-approach-antibody-validation-principles/antibody-validation-for-western-blotting/ourapproach-validation-western-blot

B) The anti-GHRHR antibody (from LSBio, Cat. No LS-B6566) is Validated for IF, IHC, Peptide-ELISA and WB. It was also tested on 20 paraffin-embedded human tissues.

https://www.lsbio.com/antibodies/ihc-plus-ghrhr-antibody-aa351-400-if-immunofluorescence-ihc-wb-western-ls-b6566/154304#validation-section

C) The anti-Pit1 antibody (Cat. No NBP1-92273) is Validated by « Independent antibodies » -Novus Biologicals. Here are the pillars of the antibody validation at Novus Biologicals :
https://www.novusbio.com/products/pit1-antibody_nbp1-92273
https://www.novusbio.com/5-pillars-validation

D) All the antibodies coming from Sigma-Aldrich or Merck Millipore (anti-Ki-67, Cat. No AB9260) were validated :
https://www.merckmillipore.com/BE/fr/life-science-research/antibodies-assays/antibodies-overview/l6Wb.qB.p6cAAAFOKNAqQvST,nav
The monoclonal ANTI-FLAG® M2 antibody (Cat. No F3165) is a trademark and validated by Sigma-Aldrich. It is also cited in more than 4000 peer-reviewed articles:
https://www.sigmaaldrich.com/catalog/product/SIGMA/F3165?lang=fr&region=BE

E) The anti-GH goat polyclonal antibody (Cat. No AF1067-SP) was validated by R&D systems. Here is the procedure of antibody validation at R&D systems:
https://www.rndsystems.com/resources/white-paper/development-validated-monoclonal-antibodies
https://www.rndsystems.com/products/human-mouse-rat-growth-hormone-antibody_af1067

F) The antibodies (anti-SOX2, anti-PRL, …) were validated by abcam. Here are the antibody validation standards for abcam :
https://www.abcam.com/primary-antibodies/improving-reproducibility-with-better-antibodies

G) The antibodies (anti-ACTH, anti-TSH, …)coming from Bioss and antibodies online were validated :
https://www.anticorps-enligne.fr/antibody/independently-validated-antibodies/

H) Anti-LH antibody (Cat. No LH-101AP) was validated by FabGennix :
https://fabgennix.com/epages/862cc93a-7f43-4d98-8516-1e11a7720fd8.sf/en_US/?ObjectPath=/Shops/862cc93a-7f43-4d98-8516-1e11a7720fd8/Categories/Lab_Services_Forms/%22Immunohistochemistry%20and%20Imaging%20Lab%20Services%22

Eukaryotic cell lines

Policy information about cell lines

Cell line source(s)

HEK293: ATCC, Manassas, VA, USA
Rat pituitary tumour GH3 cells (Sigma-Aldrich, Cat. No 87012603)

Authentication

Cell lines were used as provided commercially and no additional identification was performed.

Mycoplasma contamination	No mycoplasma were detected during the course of the project
Commonly misidentified lines (See ICLAC register)	No commonly misidentified cell lines were used in the study.

Animals and other organisms

Policy information about studies involving animals; ARRIVE guidelines recommended for reporting animal research

Laboratory animals	All mice were bred and maintained on a C57BL/6J genetic background and were housed in standard cages under specific pathogen-free conditions (SPF) at the animal facility of the University of Liège, fed standard mouse chow and water ad libitum and kept on a 12-hour light/dark cycle. Temperatures of 18-23°C with 40-60% humidity were adopted for mouse housing in the animal facility room. The age of mice is 27-29 week old for all experiments, except:<br>- 3 weeks until 69 weeks of age: for the measurement of body weight (up to 53 weeks) and length<br>- 6, 26 and 52 weeks of age: for plasma hormone quantification<br>- 11 months of age for Glucose Tolerance tests.
Wild animals	No wild animals were used in the study.
Field-collected samples	No field collected samples were used in the study.
Ethics oversight	All experiments were approved by the animal care and use committee of the University of Liège under the accredited protocol number 1812 and 1776.

Note that full information on the approval of the study protocol must also be provided in the manuscript.

Human research participants

Policy information about studies involving human research participants

Population characteristics	All relevant covariates such as patient gender, age at diagnosis, tumor size and invasion, and germline genotype are specifically reported for each patient in Supplementary Table 2. There were no treatments or other interventions in the human subjects in this study
Recruitment	Patients were recruited under existing clinical study protocols and Ethics Committee approvals for the study of the impact of genetic mutations on pituitary tumor characteristics. We selected patients randomly with no identified genetic cause, AIP germline mutations ad X-LAG in whom tissue samples were available for study. There was no self selection bias evident.
Ethics oversight	The approval for the human tissue studies was provided by the Ethics Committee of the Centre Hospitalier Universitaire de Liège, Belgium.

Note that full information on the approval of the study protocol must also be provided in the manuscript.
