# Supplementary material for: GPR101 drives growth hormone hypersecretion and gigantism in mice via constitutive activation of Gs and Gq/11
Source: Nat Commun. 2020 Sep 21;11:4752. doi: 10.1038/s41467-020-18500-x (PMC7506554; doi:10.1038/s41467-020-18500-x)
Supplement: Supplementary file 4 — Source Data [file 41467_2020_18500_MOESM4_ESM.zip › Source Data/Source data - Supplementary Figure 1 - Panel C.pptx]

## Slide 1
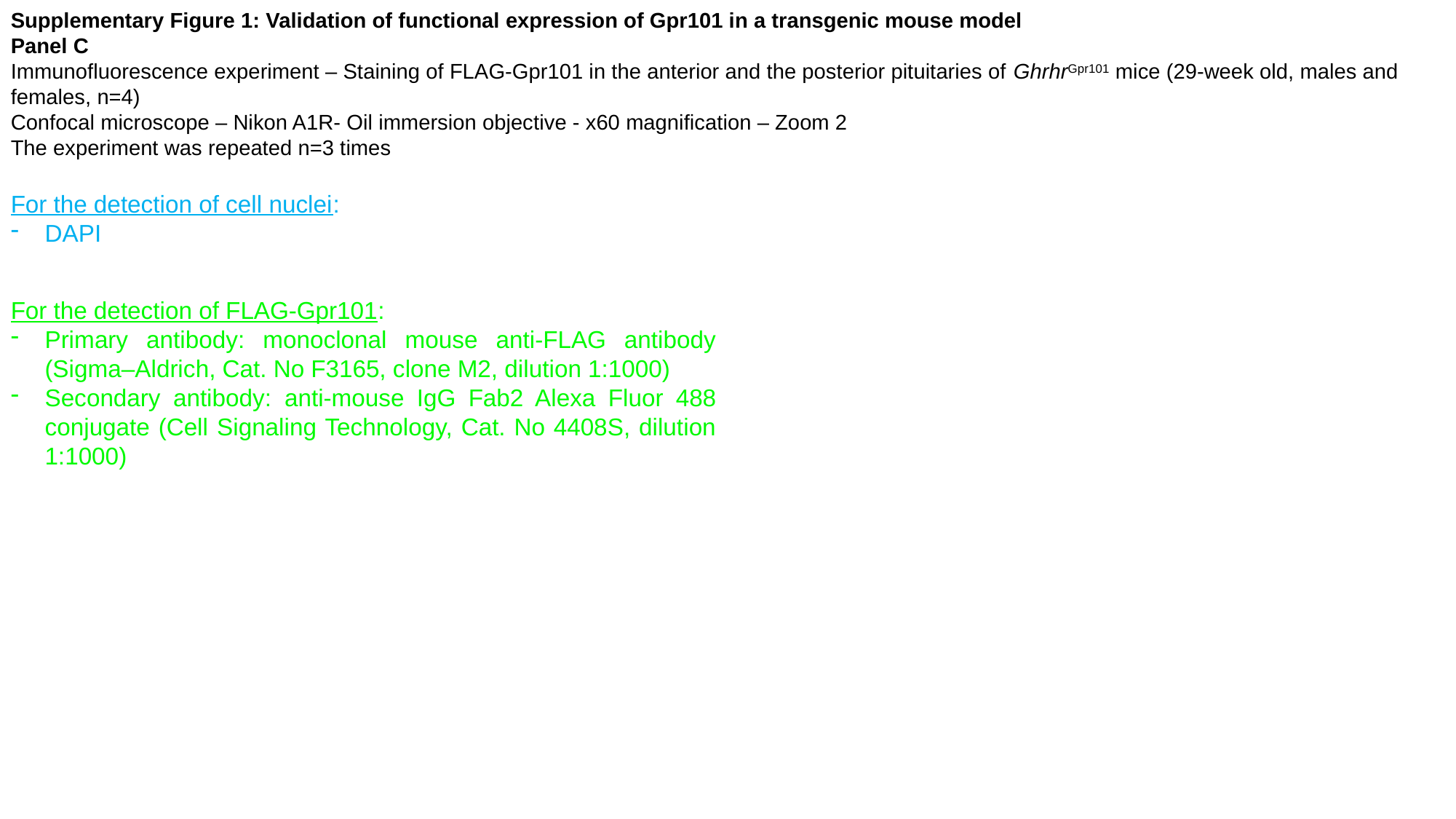

Supplementary Figure 1: Validation of functional expression of Gpr101 in a transgenic mouse model
Panel C
Immunofluorescence experiment – Staining of FLAG-Gpr101 in the anterior and the posterior pituitaries of GhrhrGpr101 mice (29-week old, males and females, n=4)
Confocal microscope – Nikon A1R- Oil immersion objective - x60 magnification – Zoom 2
The experiment was repeated n=3 times
For the detection of cell nuclei:
DAPI
For the detection of FLAG-Gpr101:
Primary antibody: monoclonal mouse anti-FLAG antibody (Sigma–Aldrich, Cat. No F3165, clone M2, dilution 1:1000)
Secondary antibody: anti-mouse IgG Fab2 Alexa Fluor 488 conjugate (Cell Signaling Technology, Cat. No 4408S, dilution 1:1000)

## Slide 2
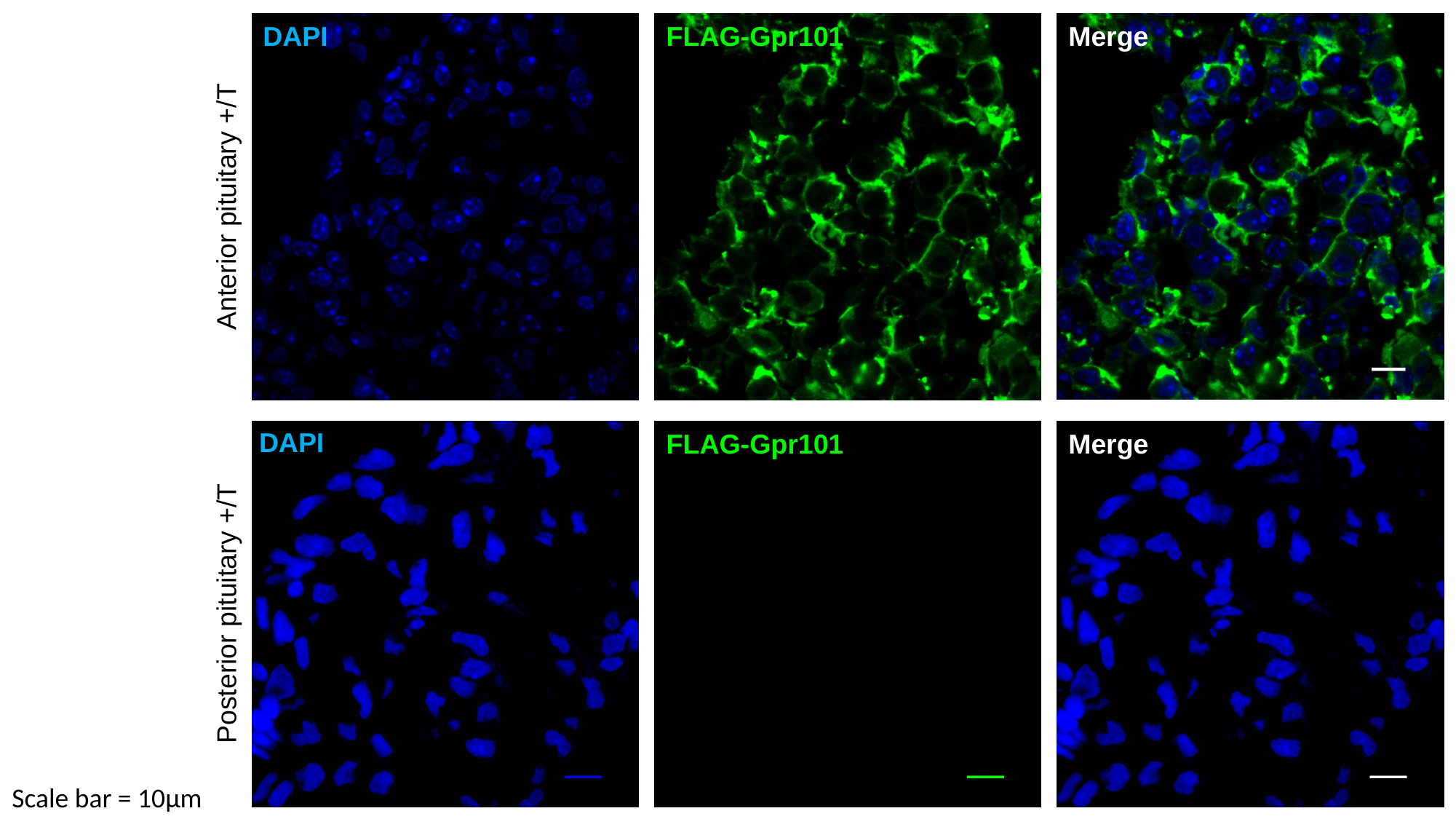

DAPI
FLAG-Gpr101
Merge
Anterior pituitary +/T
DAPI
FLAG-Gpr101
Merge
Posterior pituitary +/T
Scale bar = 10µm
