# Supplementary material for: GPR101 drives growth hormone hypersecretion and gigantism in mice via constitutive activation of Gs and Gq/11
Source: Nat Commun. 2020 Sep 21;11:4752. doi: 10.1038/s41467-020-18500-x (PMC7506554; doi:10.1038/s41467-020-18500-x)
Supplement: Supplementary file 4 — Source Data [file 41467_2020_18500_MOESM4_ESM.zip › Source Data/Source data - Supplementary Figure 1 - Panel B.pptx]

## Slide 1
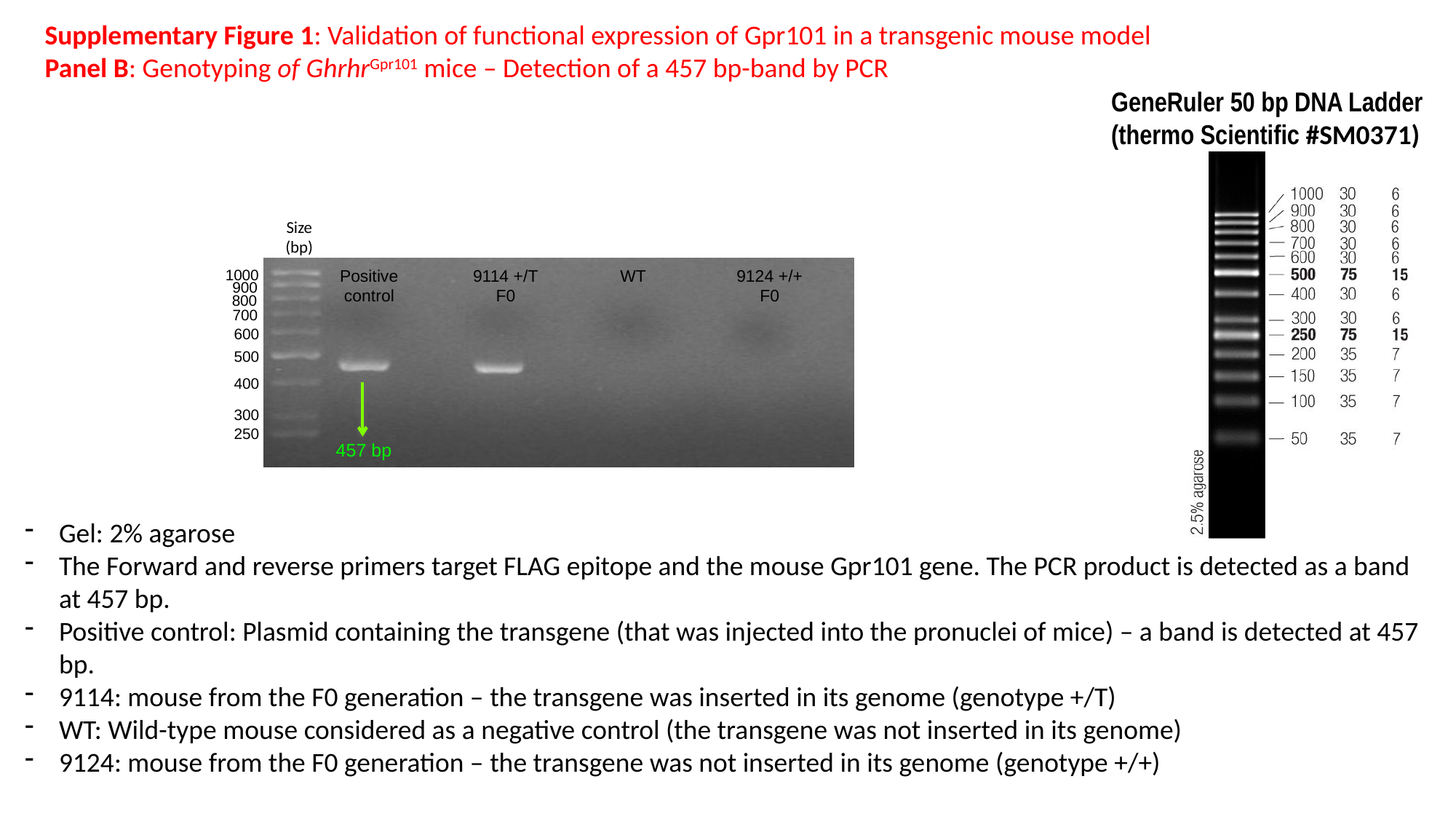

Supplementary Figure 1: Validation of functional expression of Gpr101 in a transgenic mouse model
Panel B: Genotyping of GhrhrGpr101 mice – Detection of a 457 bp-band by PCR
GeneRuler 50 bp DNA Ladder
(thermo Scientific #SM0371)
Size
(bp)
1000
9114 +/T
F0
9124 +/+
F0
Positive
control
WT
900
800
700
600
500
400
300
250
457 bp
Gel: 2% agarose
The Forward and reverse primers target FLAG epitope and the mouse Gpr101 gene. The PCR product is detected as a band at 457 bp.
Positive control: Plasmid containing the transgene (that was injected into the pronuclei of mice) – a band is detected at 457 bp.
9114: mouse from the F0 generation – the transgene was inserted in its genome (genotype +/T)
WT: Wild-type mouse considered as a negative control (the transgene was not inserted in its genome)
9124: mouse from the F0 generation – the transgene was not inserted in its genome (genotype +/+)
