# Supplementary material for: GPR101 drives growth hormone hypersecretion and gigantism in mice via constitutive activation of Gs and Gq/11
Source: Nat Commun. 2020 Sep 21;11:4752. doi: 10.1038/s41467-020-18500-x (PMC7506554; doi:10.1038/s41467-020-18500-x)
Supplement: Supplementary file 4 — Source Data [file 41467_2020_18500_MOESM4_ESM.zip › Source Data/Source data - Figure 1 - Panel K.pptx]

## Slide 1
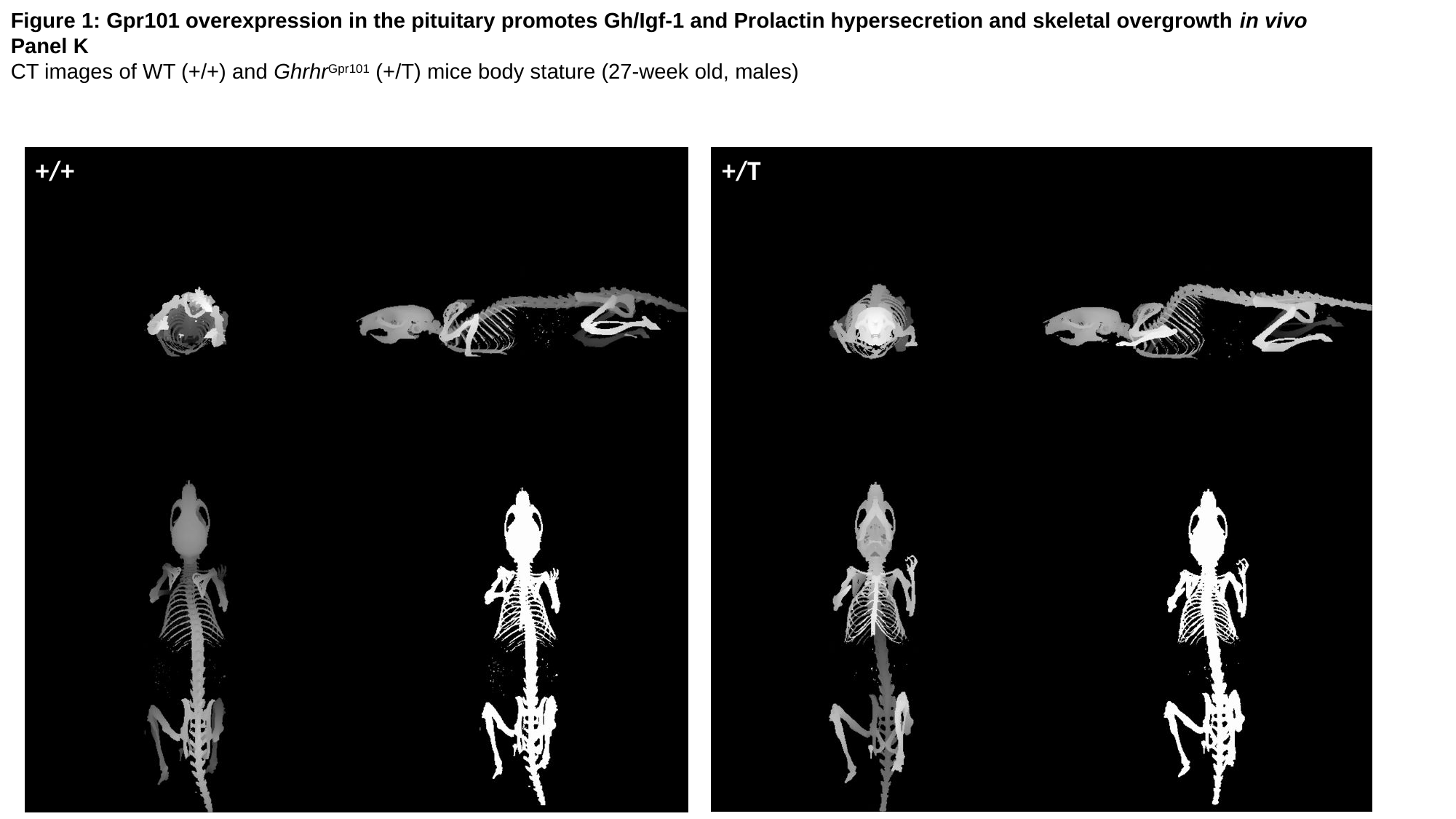

Figure 1: Gpr101 overexpression in the pituitary promotes Gh/Igf-1 and Prolactin hypersecretion and skeletal overgrowth in vivo
Panel K
CT images of WT (+/+) and GhrhrGpr101 (+/T) mice body stature (27-week old, males)
+/+
+/T

## Slide 2
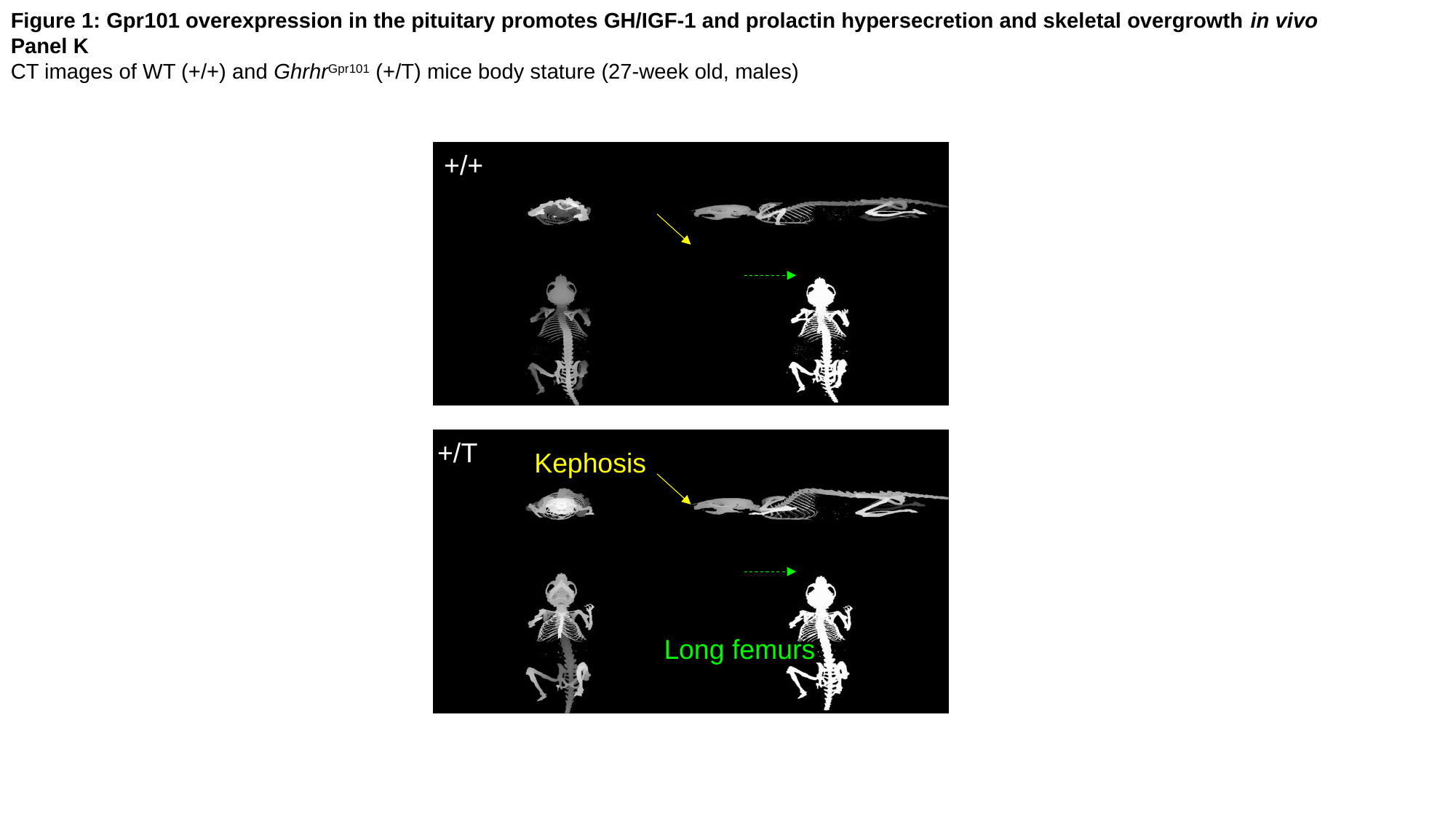

Figure 1: Gpr101 overexpression in the pituitary promotes GH/IGF-1 and prolactin hypersecretion and skeletal overgrowth in vivo
Panel K
CT images of WT (+/+) and GhrhrGpr101 (+/T) mice body stature (27-week old, males)
+/+
+/T
Kephosis
Long femurs
