# Supplementary material for: GPR101 drives growth hormone hypersecretion and gigantism in mice via constitutive activation of Gs and Gq/11
Source: Nat Commun. 2020 Sep 21;11:4752. doi: 10.1038/s41467-020-18500-x (PMC7506554; doi:10.1038/s41467-020-18500-x)
Supplement: Supplementary file 4 — Source Data [file 41467_2020_18500_MOESM4_ESM.zip › Source Data/Source data - Figure 1 - Panel J.pptx]

## Slide 1
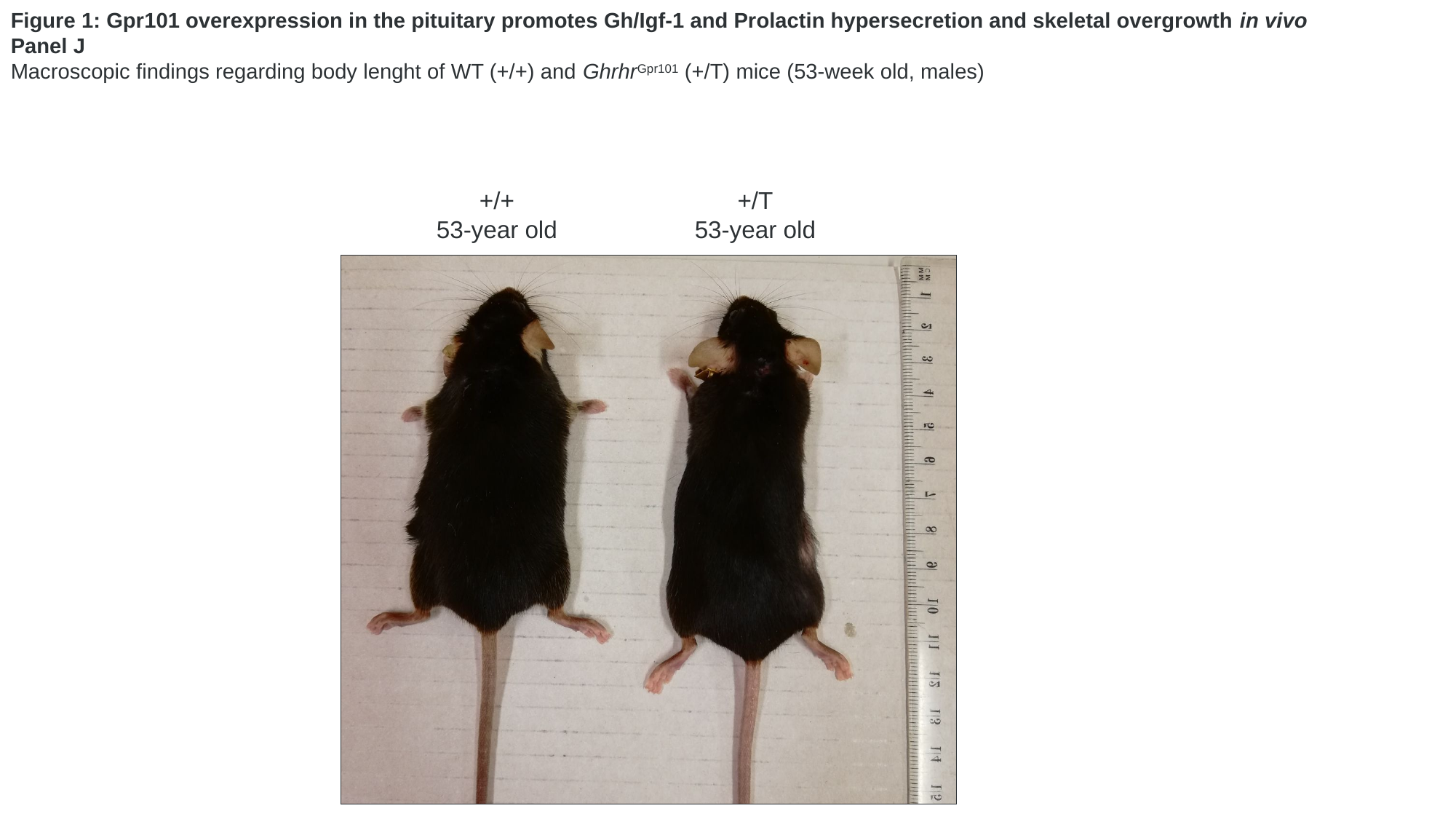

Figure 1: Gpr101 overexpression in the pituitary promotes Gh/Igf-1 and Prolactin hypersecretion and skeletal overgrowth in vivo
Panel J
Macroscopic findings regarding body lenght of WT (+/+) and GhrhrGpr101 (+/T) mice (53-week old, males)
+/+
53-year old
+/T
53-year old
