# Supplementary material for: GPR101 drives growth hormone hypersecretion and gigantism in mice via constitutive activation of Gs and Gq/11
Source: Nat Commun. 2020 Sep 21;11:4752. doi: 10.1038/s41467-020-18500-x (PMC7506554; doi:10.1038/s41467-020-18500-x)
Supplement: Supplementary file 4 — Source Data [file 41467_2020_18500_MOESM4_ESM.zip › Source Data/Source data - Figure 3 - Panel A.pptx]

## Slide 1
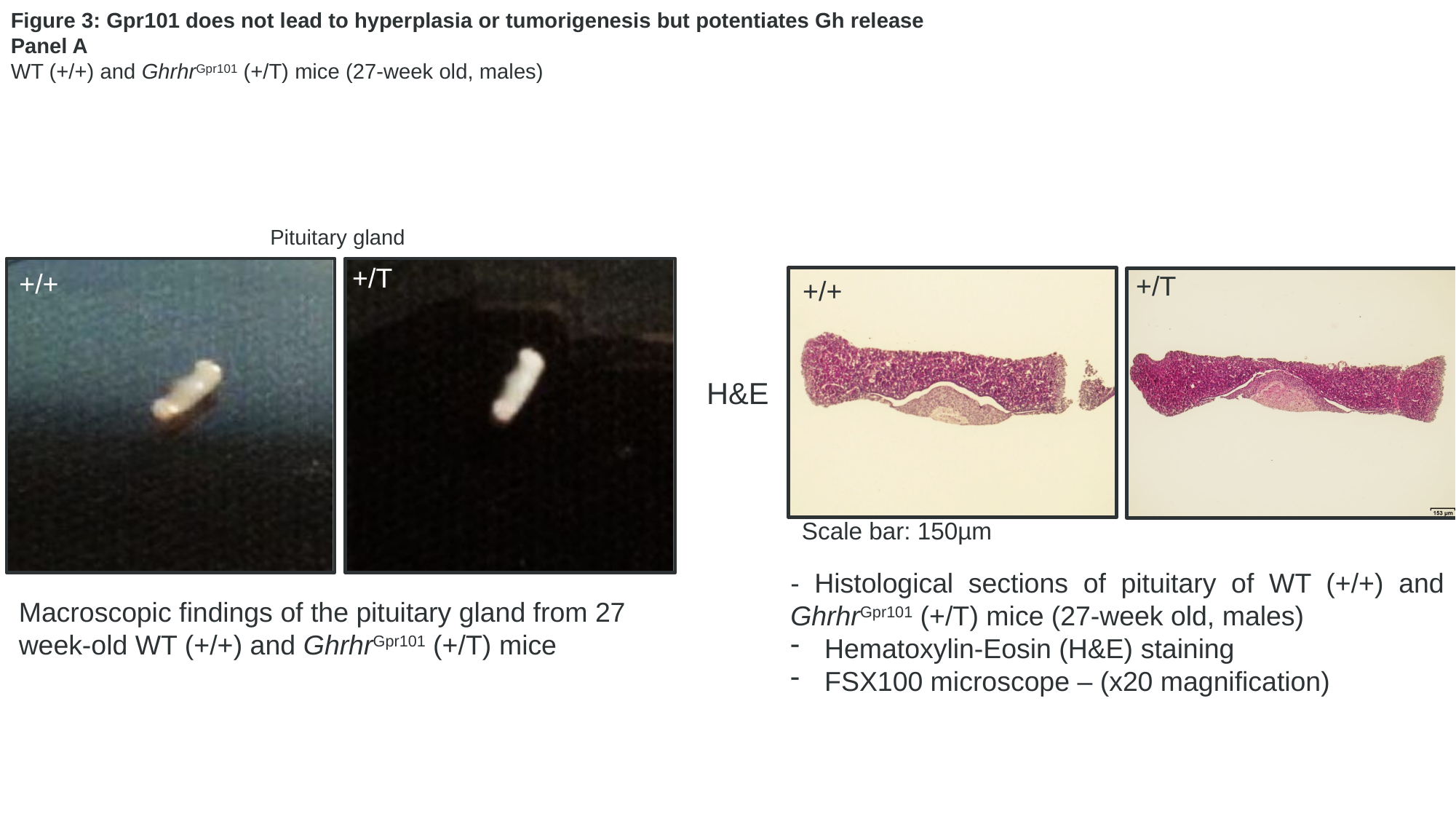

Figure 3: Gpr101 does not lead to hyperplasia or tumorigenesis but potentiates Gh release
Panel A
WT (+/+) and GhrhrGpr101 (+/T) mice (27-week old, males)
Pituitary gland
+/T
+/+
+/T
+/+
H&E
Scale bar: 150µm
- Histological sections of pituitary of WT (+/+) and GhrhrGpr101 (+/T) mice (27-week old, males)
Hematoxylin-Eosin (H&E) staining
FSX100 microscope – (x20 magnification)
Macroscopic findings of the pituitary gland from 27 week-old WT (+/+) and GhrhrGpr101 (+/T) mice
