# Supplementary material for: GPR101 drives growth hormone hypersecretion and gigantism in mice via constitutive activation of Gs and Gq/11
Source: Nat Commun. 2020 Sep 21;11:4752. doi: 10.1038/s41467-020-18500-x (PMC7506554; doi:10.1038/s41467-020-18500-x)
Supplement: Supplementary file 4 — Source Data [file 41467_2020_18500_MOESM4_ESM.zip › Source Data/Source data - Supplementary Figure 1 - Panel H.pptx]

## Slide 1
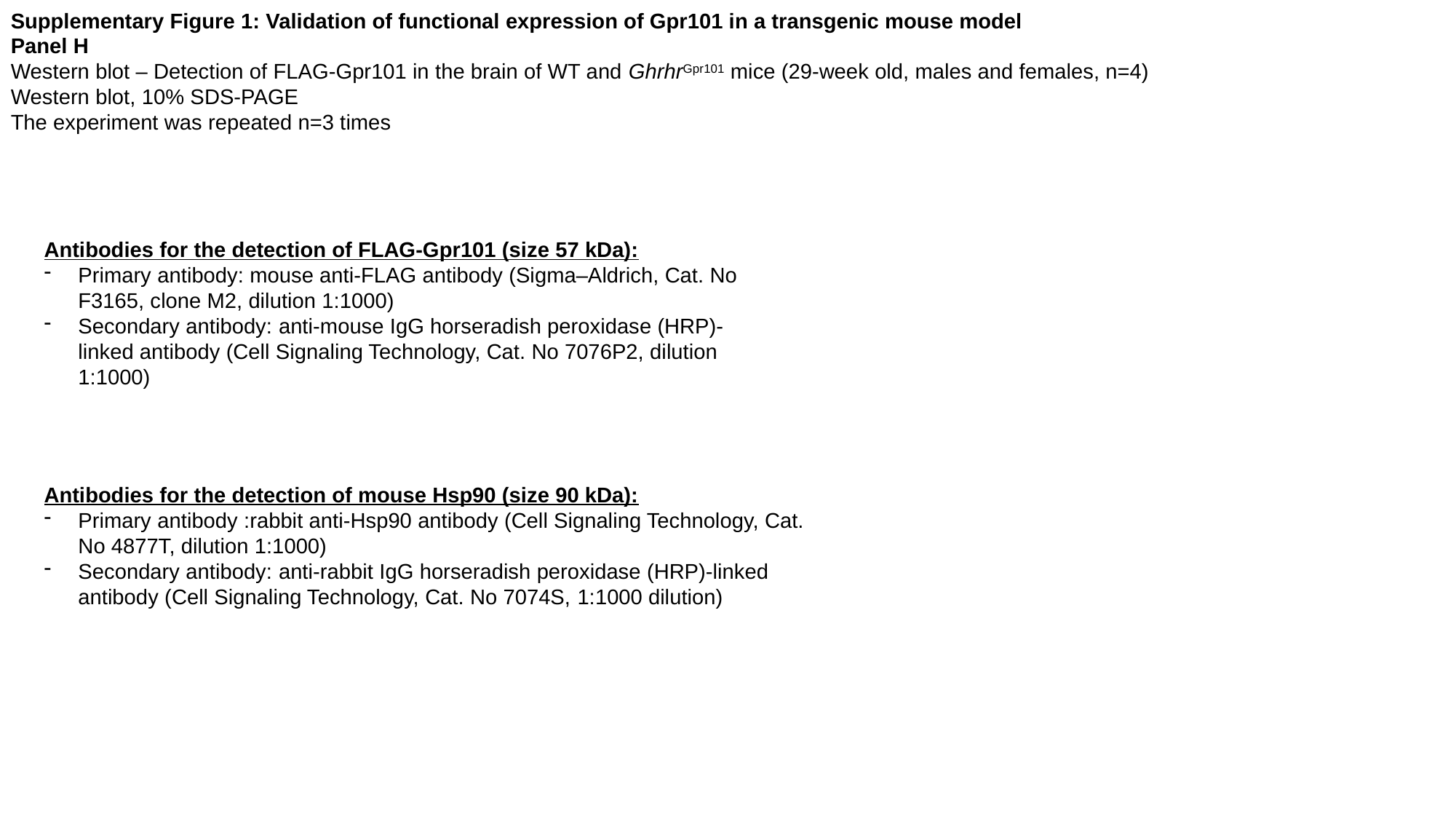

Supplementary Figure 1: Validation of functional expression of Gpr101 in a transgenic mouse model
Panel H
Western blot – Detection of FLAG-Gpr101 in the brain of WT and GhrhrGpr101 mice (29-week old, males and females, n=4)
Western blot, 10% SDS-PAGE
The experiment was repeated n=3 times
Antibodies for the detection of FLAG-Gpr101 (size 57 kDa):
Primary antibody: mouse anti-FLAG antibody (Sigma–Aldrich, Cat. No F3165, clone M2, dilution 1:1000)
Secondary antibody: anti-mouse IgG horseradish peroxidase (HRP)-linked antibody (Cell Signaling Technology, Cat. No 7076P2, dilution 1:1000)
Antibodies for the detection of mouse Hsp90 (size 90 kDa):
Primary antibody :rabbit anti-Hsp90 antibody (Cell Signaling Technology, Cat. No 4877T, dilution 1:1000)
Secondary antibody: anti-rabbit IgG horseradish peroxidase (HRP)-linked antibody (Cell Signaling Technology, Cat. No 7074S, 1:1000 dilution)

## Slide 2
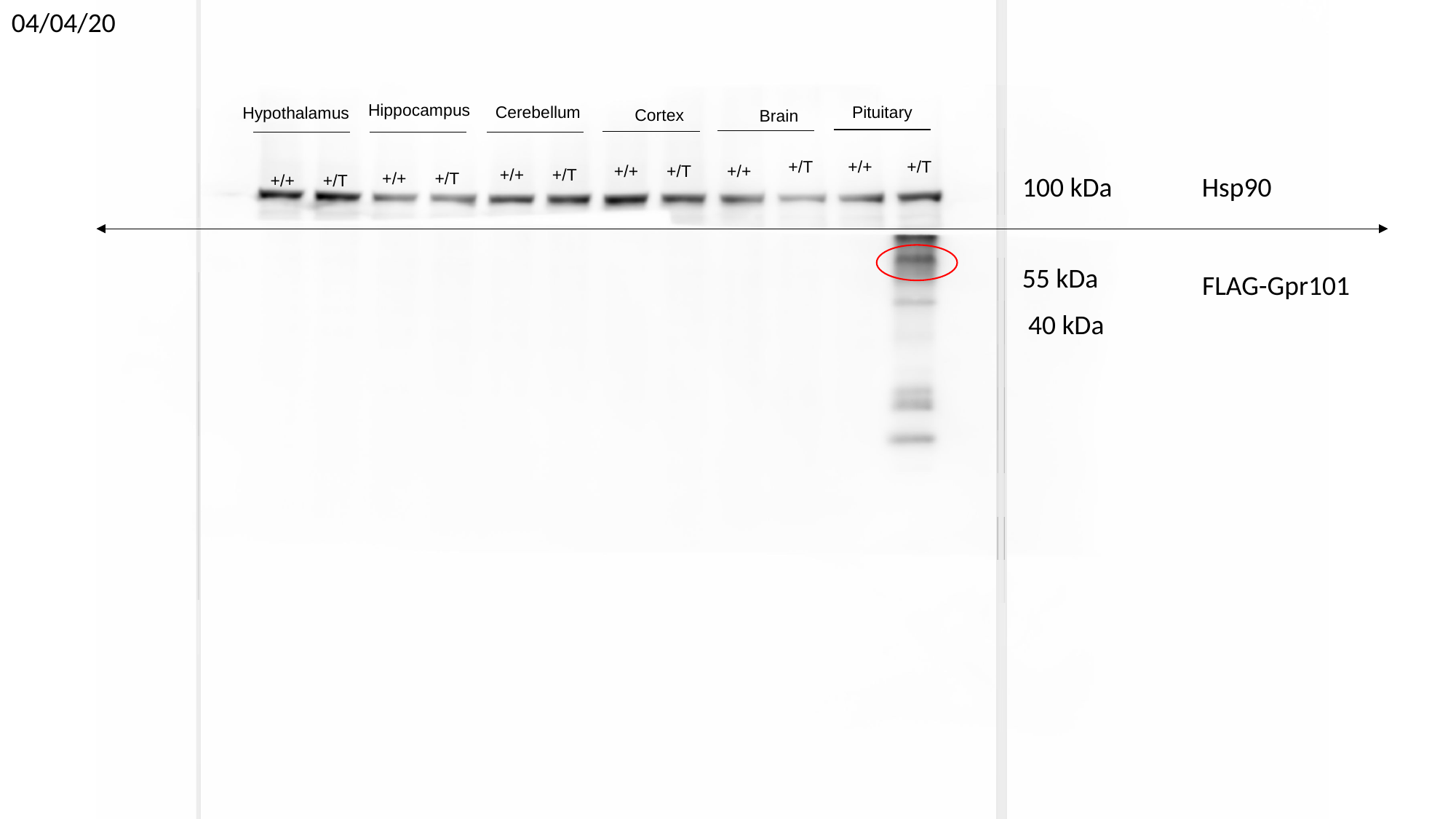

04/04/20
Hippocampus
Cerebellum
Pituitary
Hypothalamus
Cortex
Brain
+/T
+/+
+/T
+/+
+/T
+/+
+/+
+/T
+/+
+/T
100 kDa
Hsp90
+/+
+/T
55 kDa
FLAG-Gpr101
40 kDa

## Slide 3
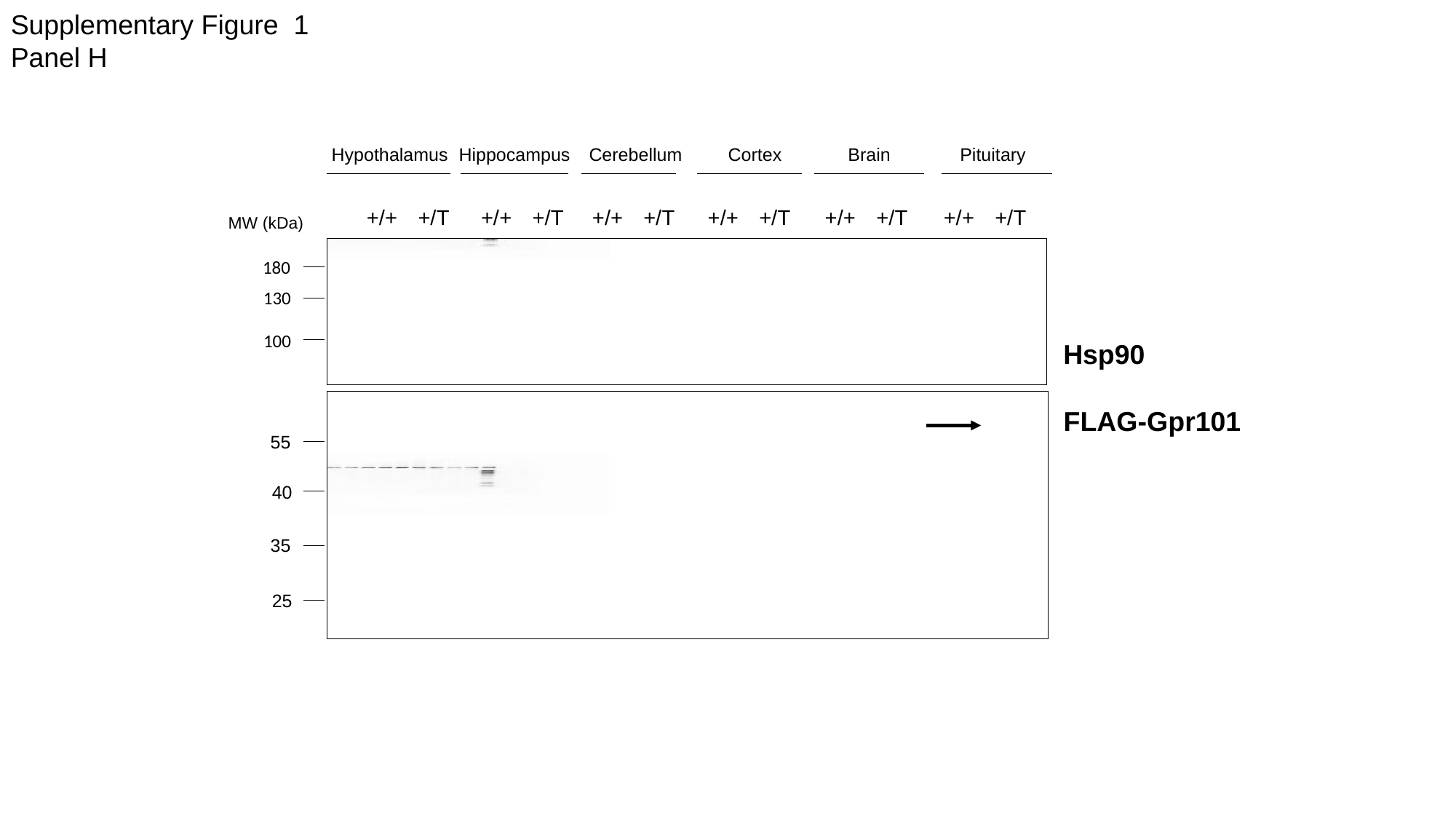

Supplementary Figure 1
Panel H
Hypothalamus
Hippocampus
Cerebellum
Cortex
Brain
Pituitary
+/+
+/T
+/+
+/T
+/+
+/T
+/+
+/T
+/+
+/T
+/+
+/T
MW (kDa)
180
130
100
Hsp90
FLAG-Gpr101
55
40
35
25
