# Supplementary material for: GPR101 drives growth hormone hypersecretion and gigantism in mice via constitutive activation of Gs and Gq/11
Source: Nat Commun. 2020 Sep 21;11:4752. doi: 10.1038/s41467-020-18500-x (PMC7506554; doi:10.1038/s41467-020-18500-x)
Supplement: Supplementary file 4 — Source Data [file 41467_2020_18500_MOESM4_ESM.zip › Source Data/Source data - Figure 7 - Panel D.pptx]

## Slide 1
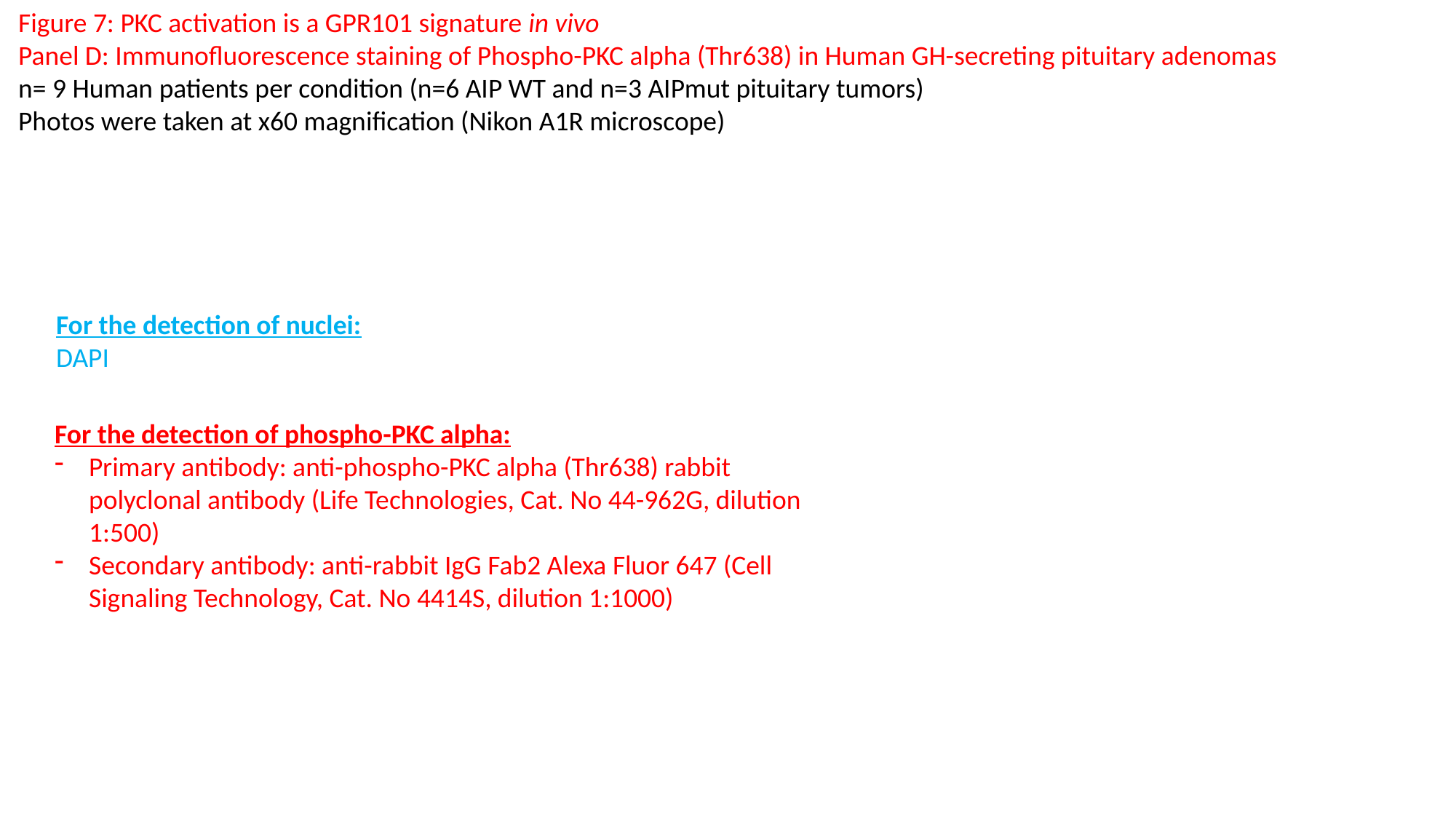

Figure 7: PKC activation is a GPR101 signature in vivo
Panel D: Immunofluorescence staining of Phospho-PKC alpha (Thr638) in Human GH-secreting pituitary adenomas
n= 9 Human patients per condition (n=6 AIP WT and n=3 AIPmut pituitary tumors)
Photos were taken at x60 magnification (Nikon A1R microscope)
For the detection of nuclei:
DAPI
For the detection of phospho-PKC alpha:
Primary antibody: anti-phospho-PKC alpha (Thr638) rabbit polyclonal antibody (Life Technologies, Cat. No 44-962G, dilution 1:500)
Secondary antibody: anti-rabbit IgG Fab2 Alexa Fluor 647 (Cell Signaling Technology, Cat. No 4414S, dilution 1:1000)

## Slide 2
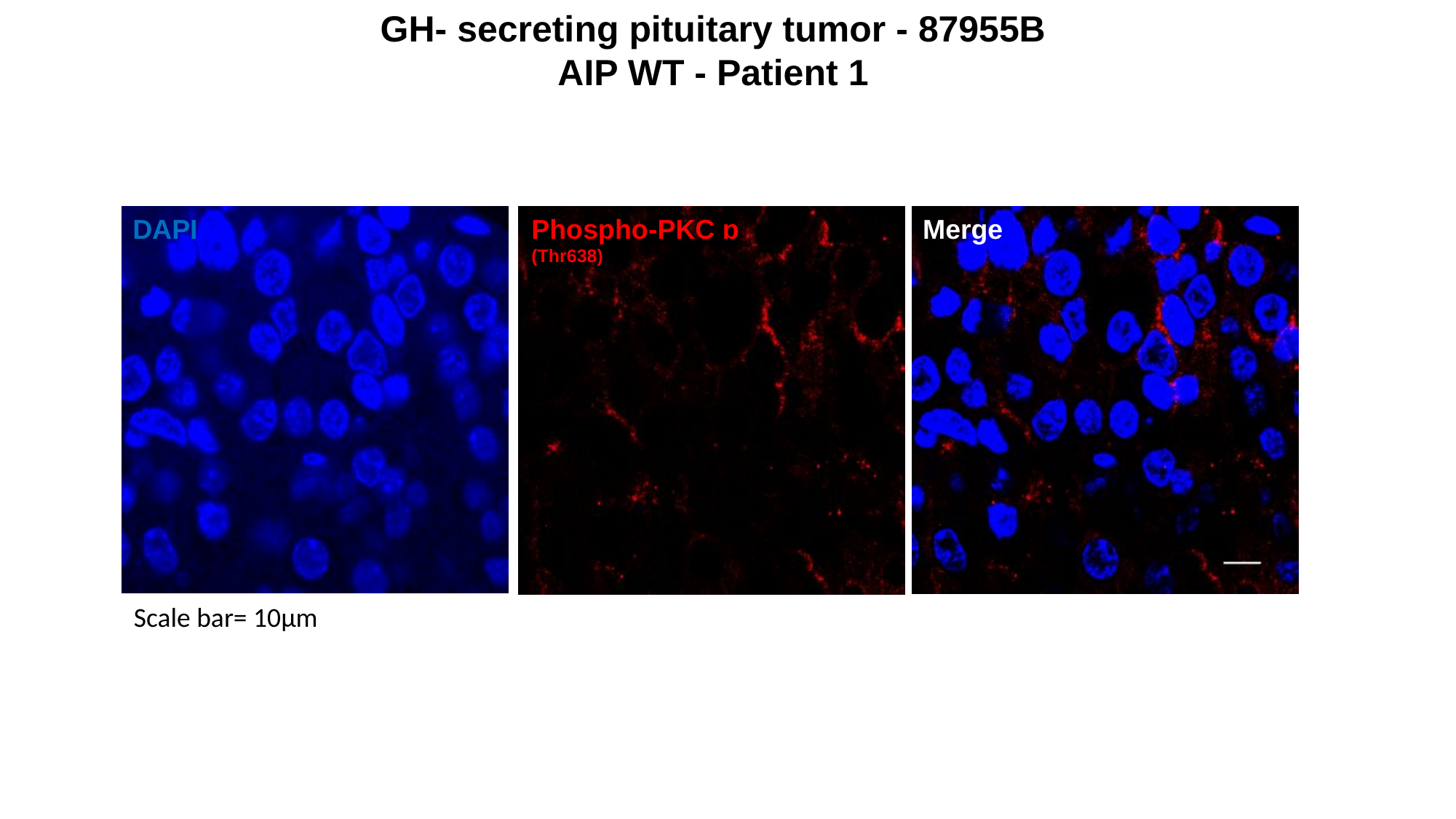

GH- secreting pituitary tumor - 87955B
AIP WT - Patient 1
DAPI
Phospho-PKC ɒ
(Thr638)
Merge
Scale bar= 10µm

## Slide 3
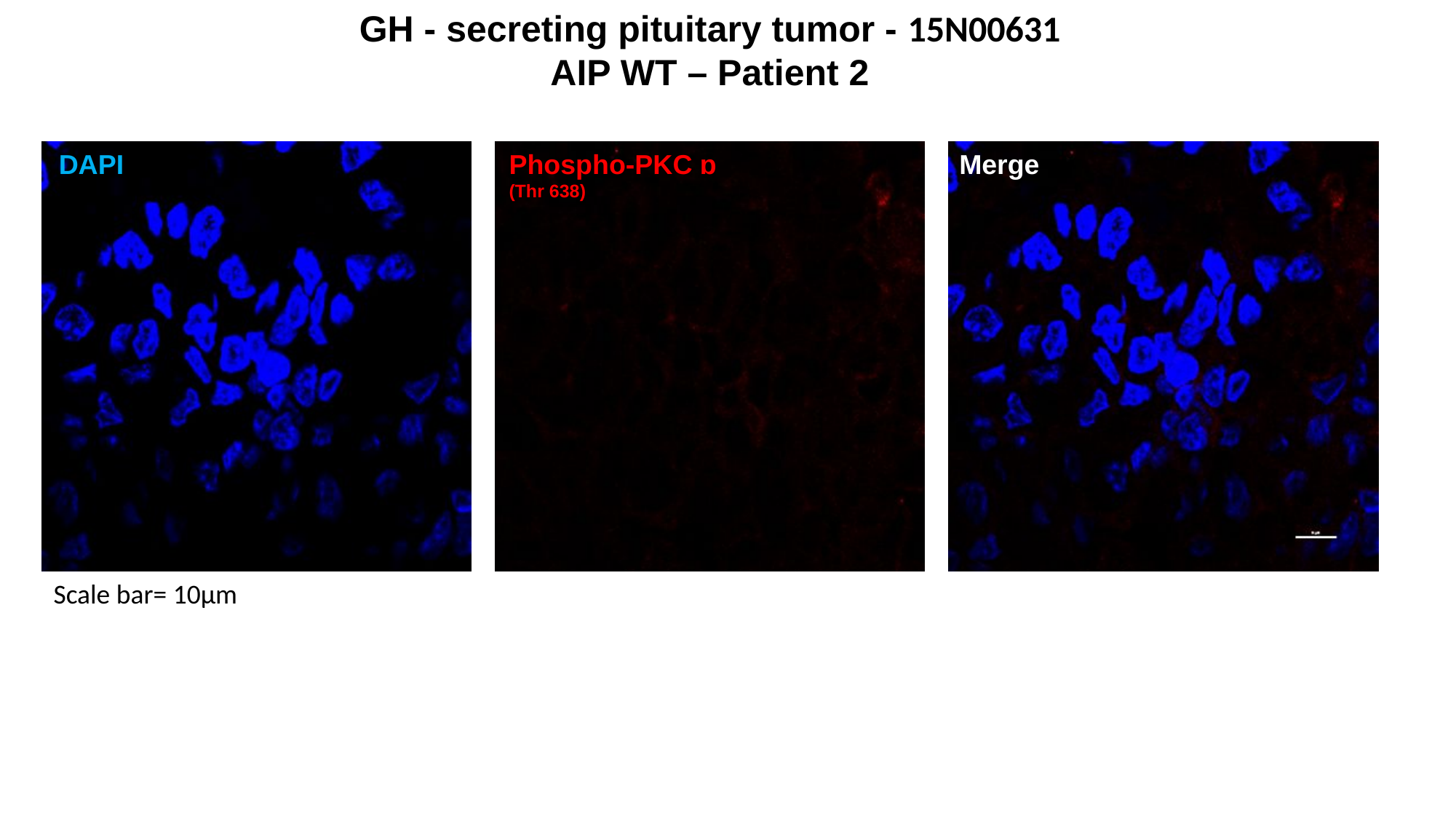

GH - secreting pituitary tumor - 15N00631
AIP WT – Patient 2
DAPI
Phospho-PKC ɒ
(Thr 638)
Merge
Scale bar= 10µm

## Slide 4
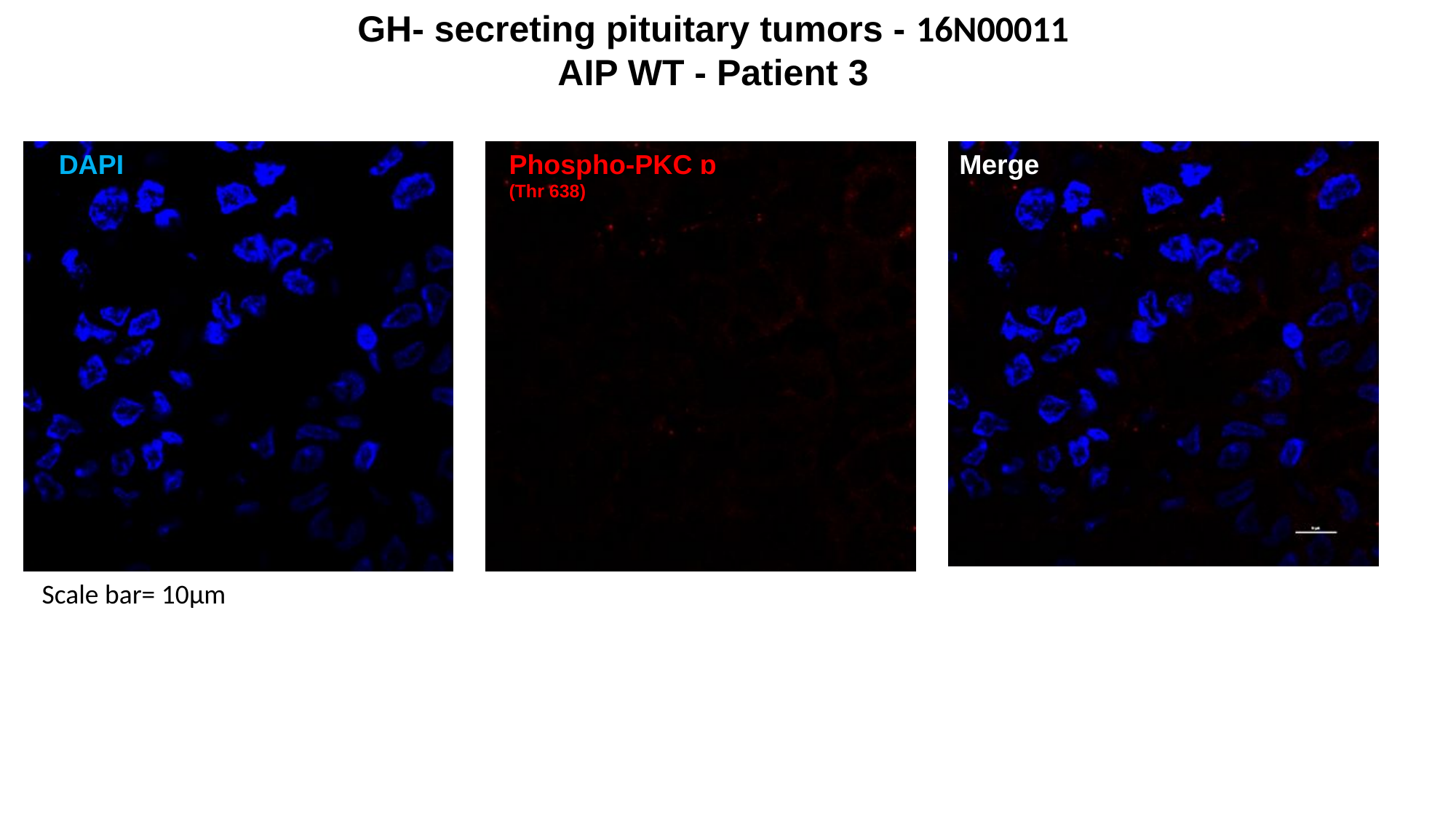

GH- secreting pituitary tumors - 16N00011
AIP WT - Patient 3
DAPI
Phospho-PKC ɒ
(Thr 638)
Merge
Scale bar= 10µm

## Slide 5
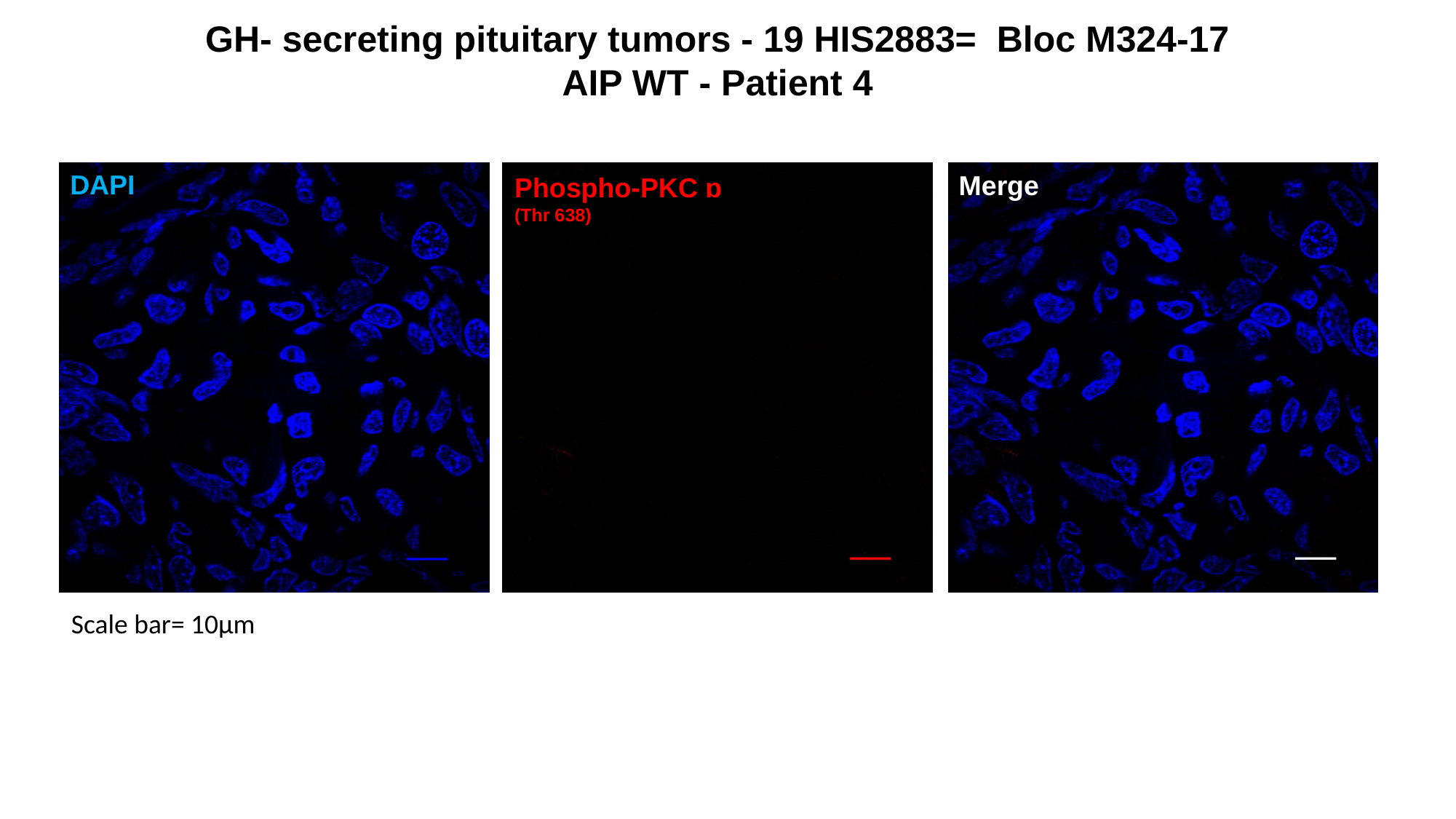

GH- secreting pituitary tumors - 19 HIS2883= Bloc M324-17
AIP WT - Patient 4
DAPI
Merge
Phospho-PKC ɒ
(Thr 638)
Scale bar= 10µm

## Slide 6
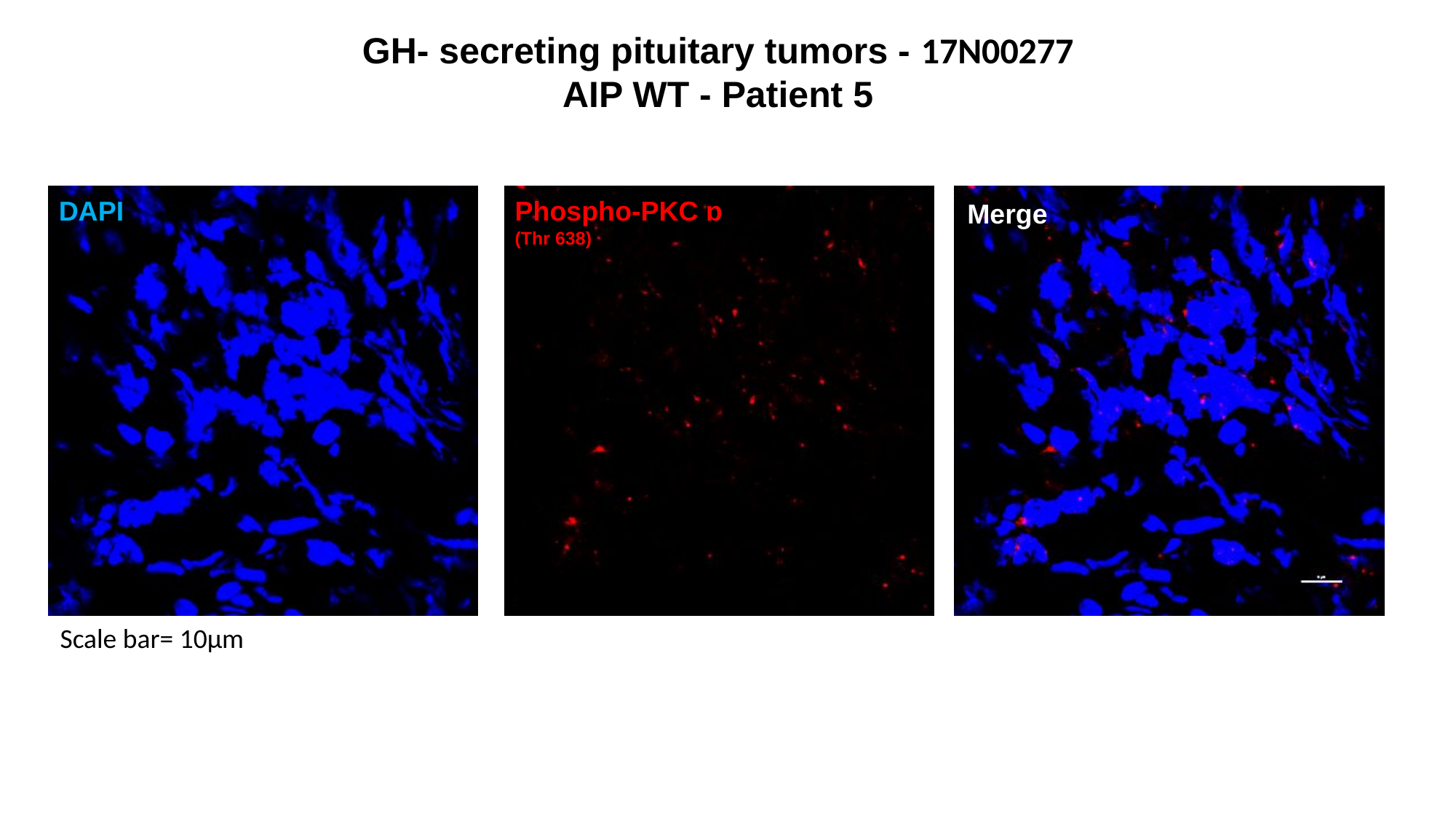

GH- secreting pituitary tumors - 17N00277
AIP WT - Patient 5
DAPI
Phospho-PKC ɒ
(Thr 638)
Merge
Scale bar= 10µm

## Slide 7
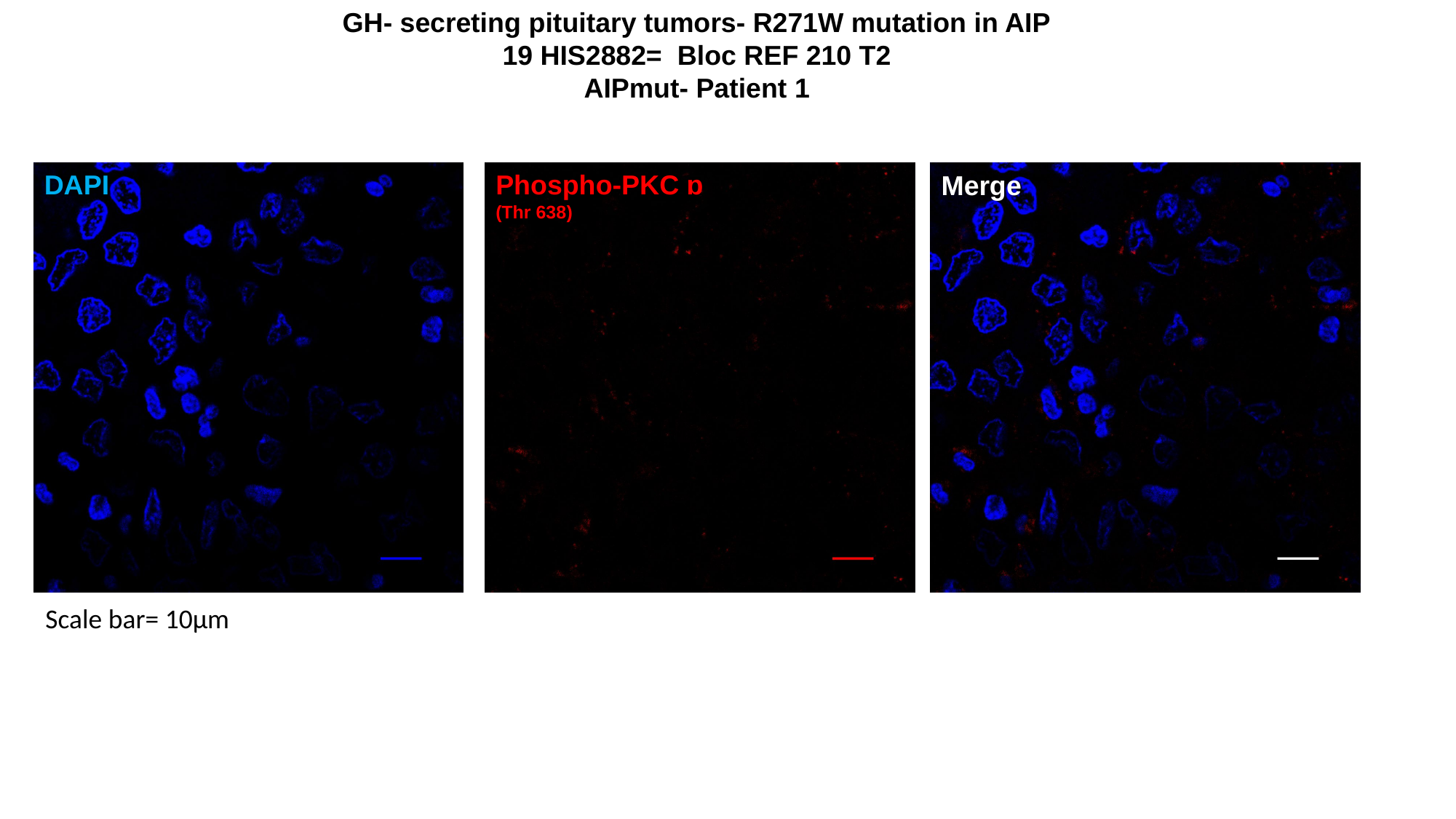

GH- secreting pituitary tumors- R271W mutation in AIP
19 HIS2882= Bloc REF 210 T2
AIPmut- Patient 1
DAPI
Phospho-PKC ɒ
(Thr 638)
Merge
Scale bar= 10µm

## Slide 8
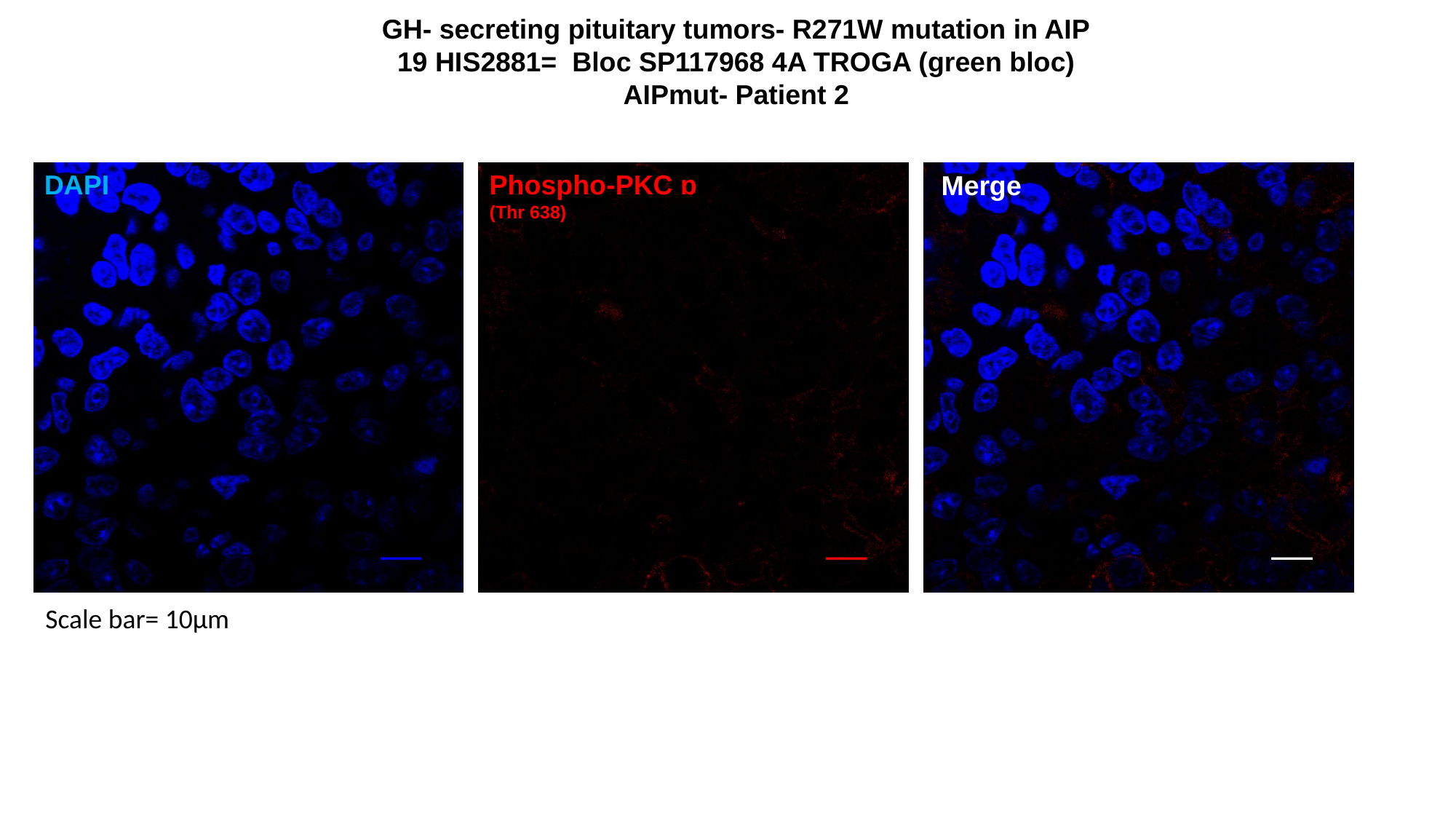

GH- secreting pituitary tumors- R271W mutation in AIP
19 HIS2881= Bloc SP117968 4A TROGA (green bloc)
AIPmut- Patient 2
DAPI
Phospho-PKC ɒ
(Thr 638)
Merge
Scale bar= 10µm

## Slide 9
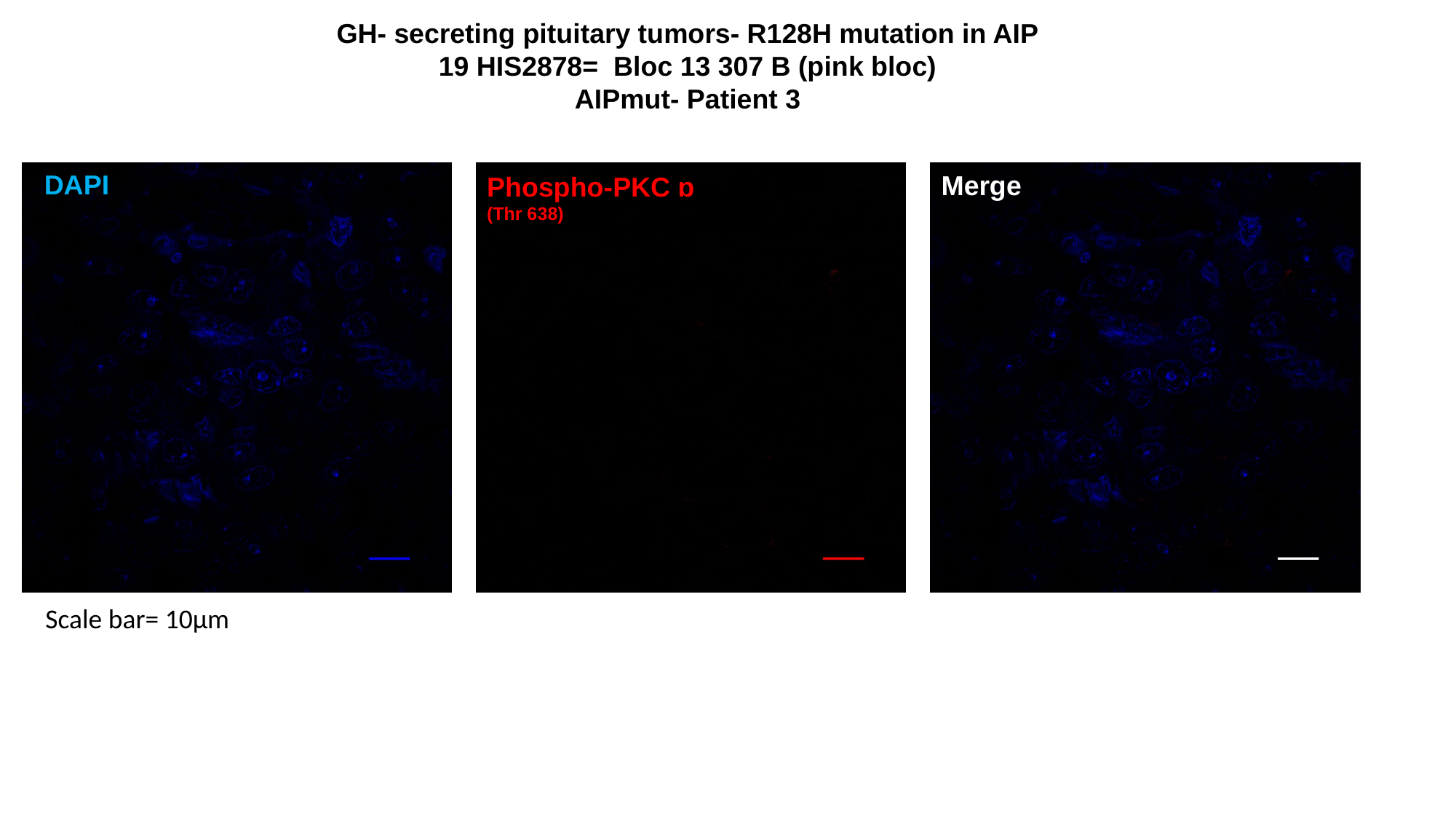

GH- secreting pituitary tumors- R128H mutation in AIP
19 HIS2878= Bloc 13 307 B (pink bloc)
AIPmut- Patient 3
DAPI
Merge
Phospho-PKC ɒ
(Thr 638)
Scale bar= 10µm

## Slide 10
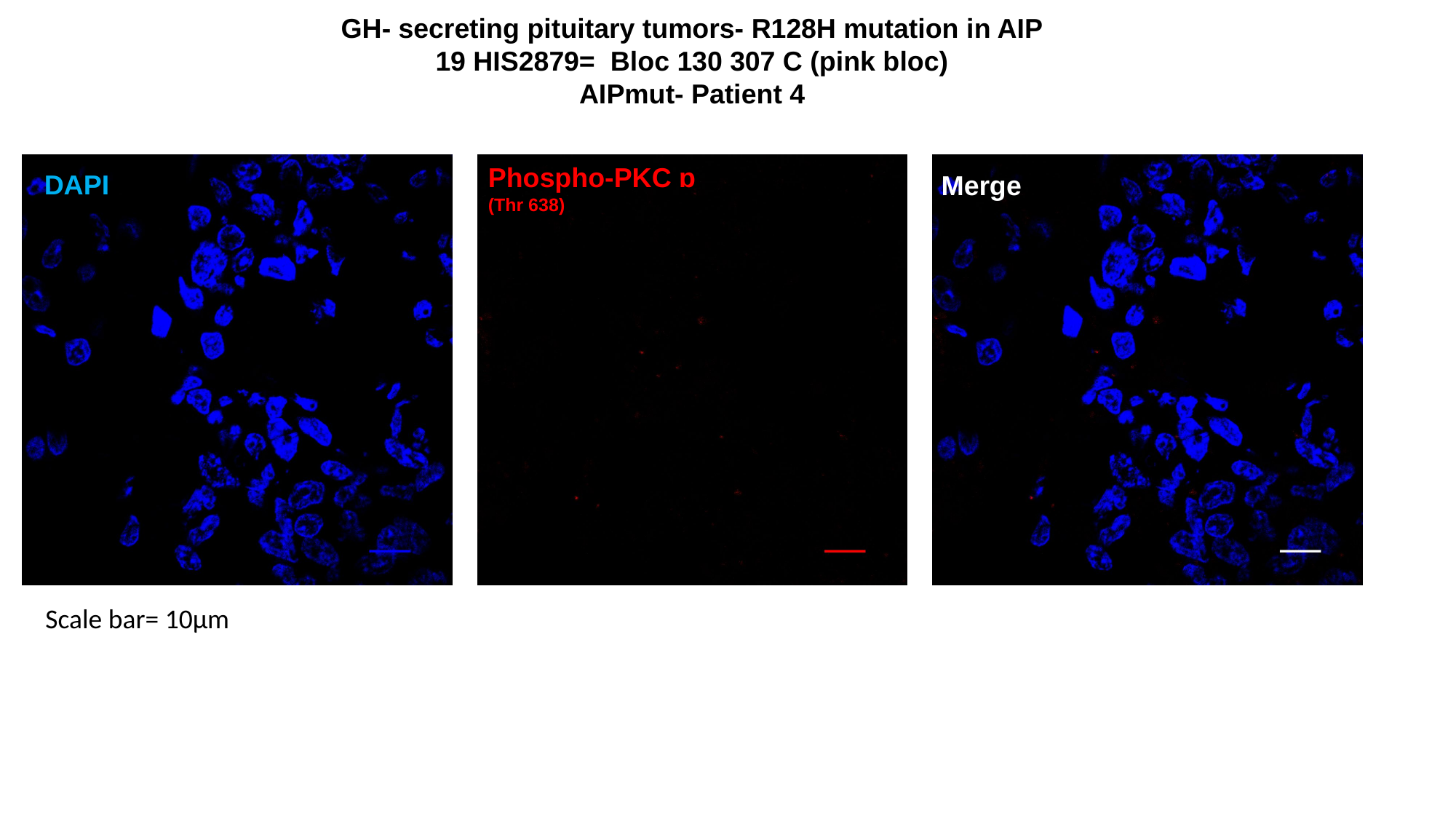

GH- secreting pituitary tumors- R128H mutation in AIP
19 HIS2879= Bloc 130 307 C (pink bloc)
AIPmut- Patient 4
Phospho-PKC ɒ
(Thr 638)
DAPI
Merge
Scale bar= 10µm

## Slide 11
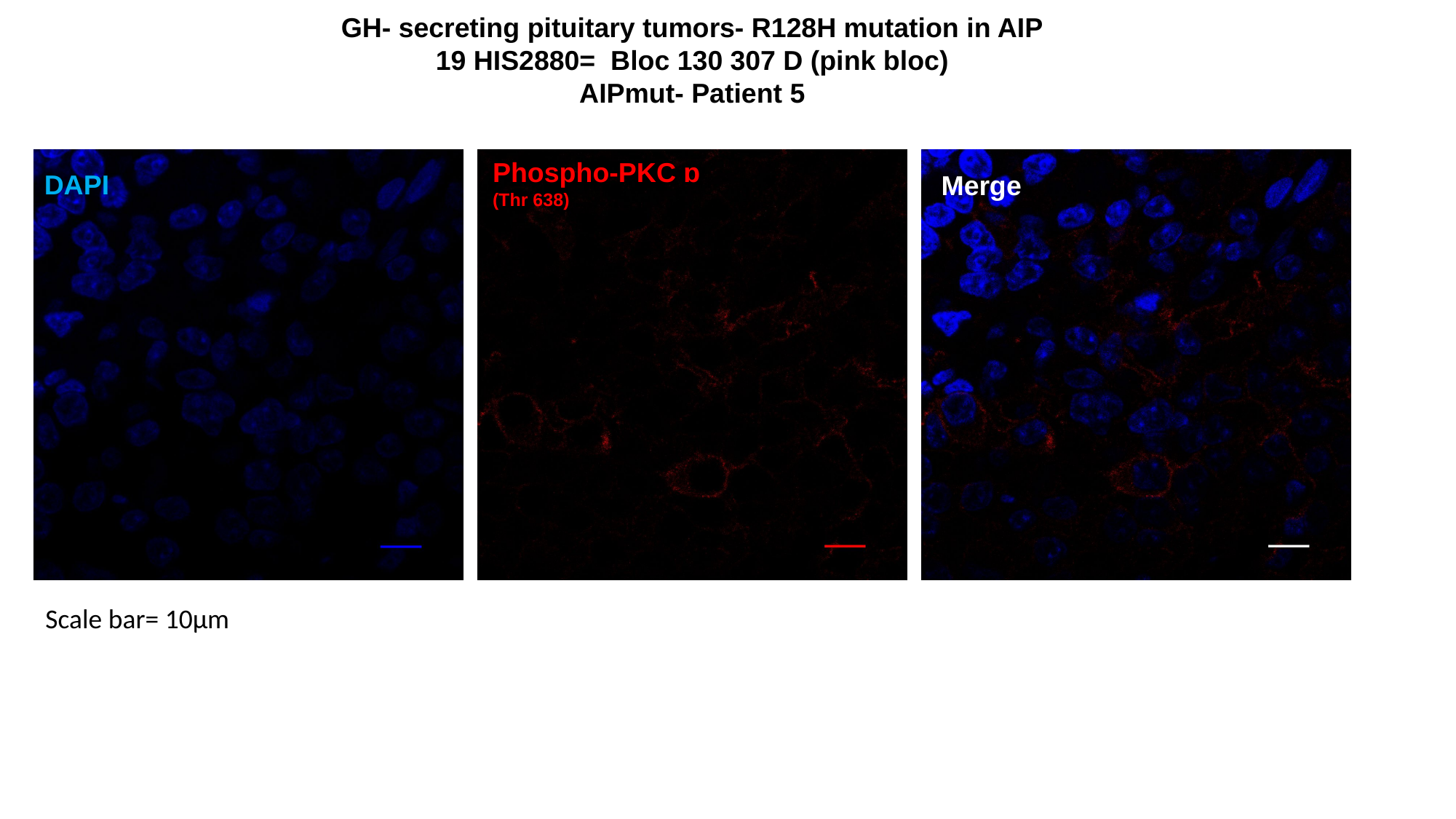

GH- secreting pituitary tumors- R128H mutation in AIP
19 HIS2880= Bloc 130 307 D (pink bloc)
AIPmut- Patient 5
Phospho-PKC ɒ
(Thr 638)
DAPI
Merge
Scale bar= 10µm
