# Supplementary material for: GPR101 drives growth hormone hypersecretion and gigantism in mice via constitutive activation of Gs and Gq/11
Source: Nat Commun. 2020 Sep 21;11:4752. doi: 10.1038/s41467-020-18500-x (PMC7506554; doi:10.1038/s41467-020-18500-x)
Supplement: Supplementary file 4 — Source Data [file 41467_2020_18500_MOESM4_ESM.zip › Source Data/Source data - Figure 7 - Panel E.pptx]

## Slide 1
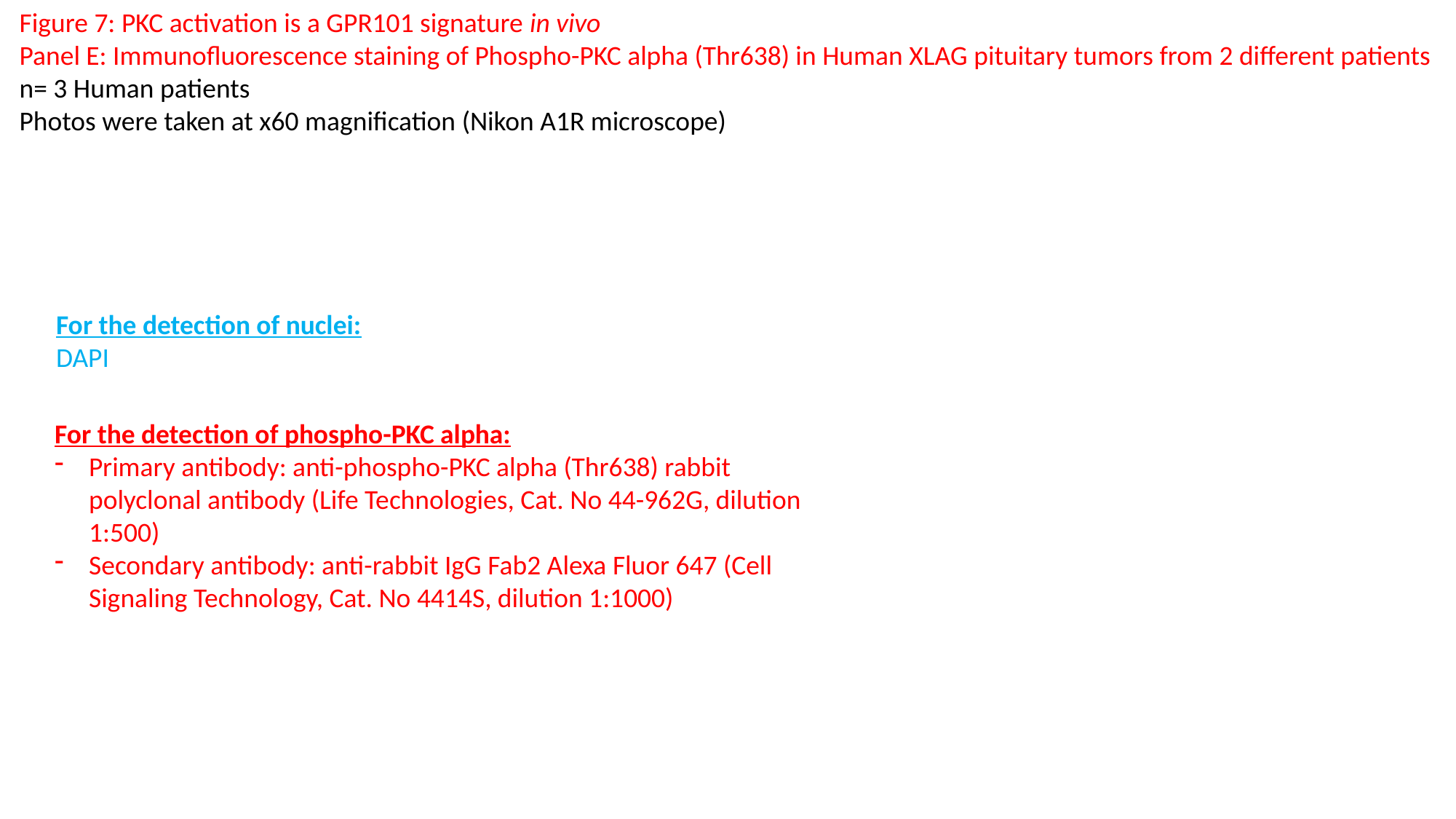

Figure 7: PKC activation is a GPR101 signature in vivo
Panel E: Immunofluorescence staining of Phospho-PKC alpha (Thr638) in Human XLAG pituitary tumors from 2 different patients
n= 3 Human patients
Photos were taken at x60 magnification (Nikon A1R microscope)
For the detection of nuclei:
DAPI
For the detection of phospho-PKC alpha:
Primary antibody: anti-phospho-PKC alpha (Thr638) rabbit polyclonal antibody (Life Technologies, Cat. No 44-962G, dilution 1:500)
Secondary antibody: anti-rabbit IgG Fab2 Alexa Fluor 647 (Cell Signaling Technology, Cat. No 4414S, dilution 1:1000)

## Slide 2
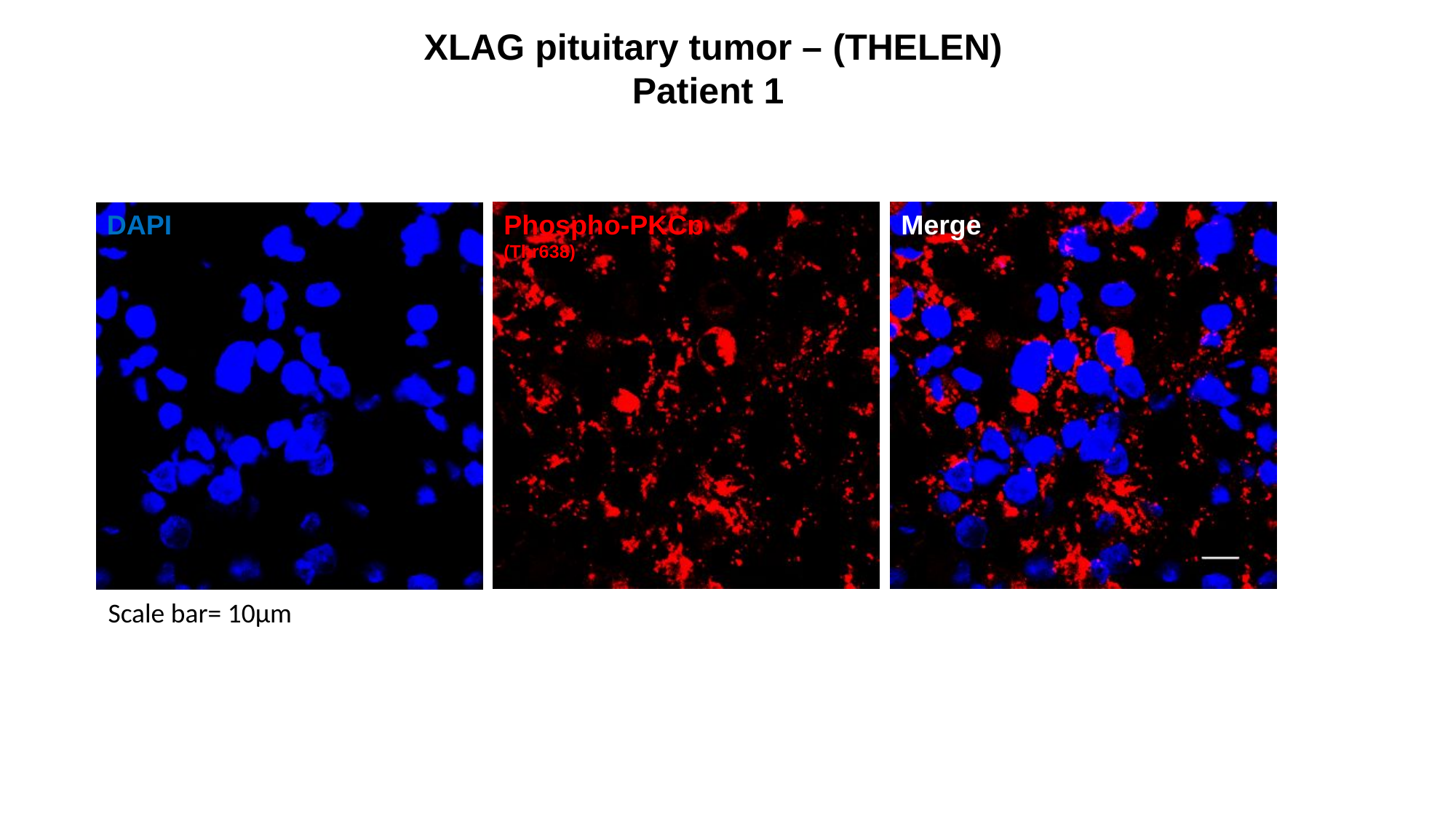

XLAG pituitary tumor – (THELEN)
Patient 1
DAPI
Phospho-PKCɒ
(Thr638)
Merge
Scale bar= 10µm

## Slide 3
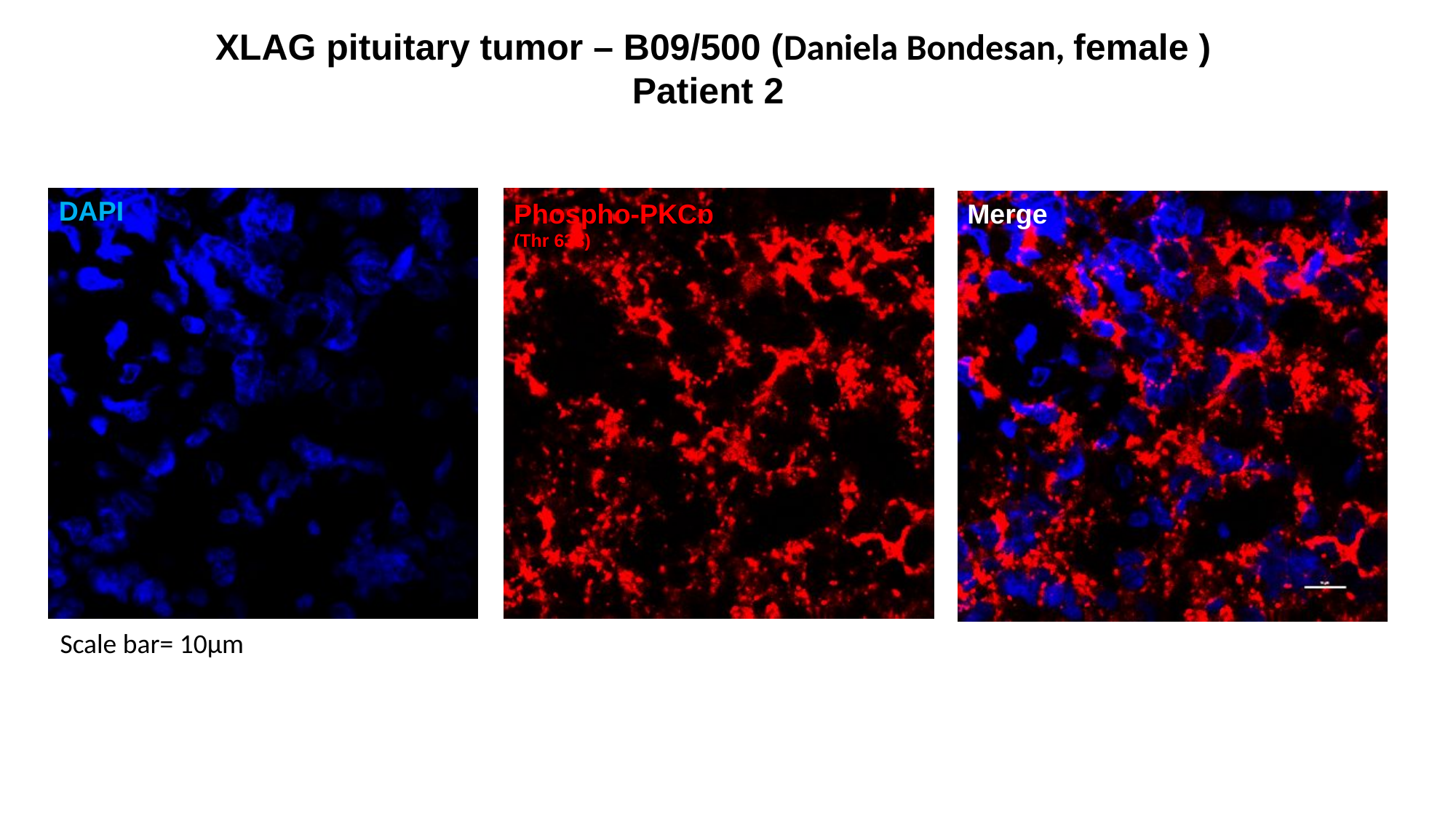

XLAG pituitary tumor – B09/500 (Daniela Bondesan, female )
Patient 2
DAPI
Phospho-PKCɒ
(Thr 638)
Merge
Scale bar= 10µm

## Slide 4
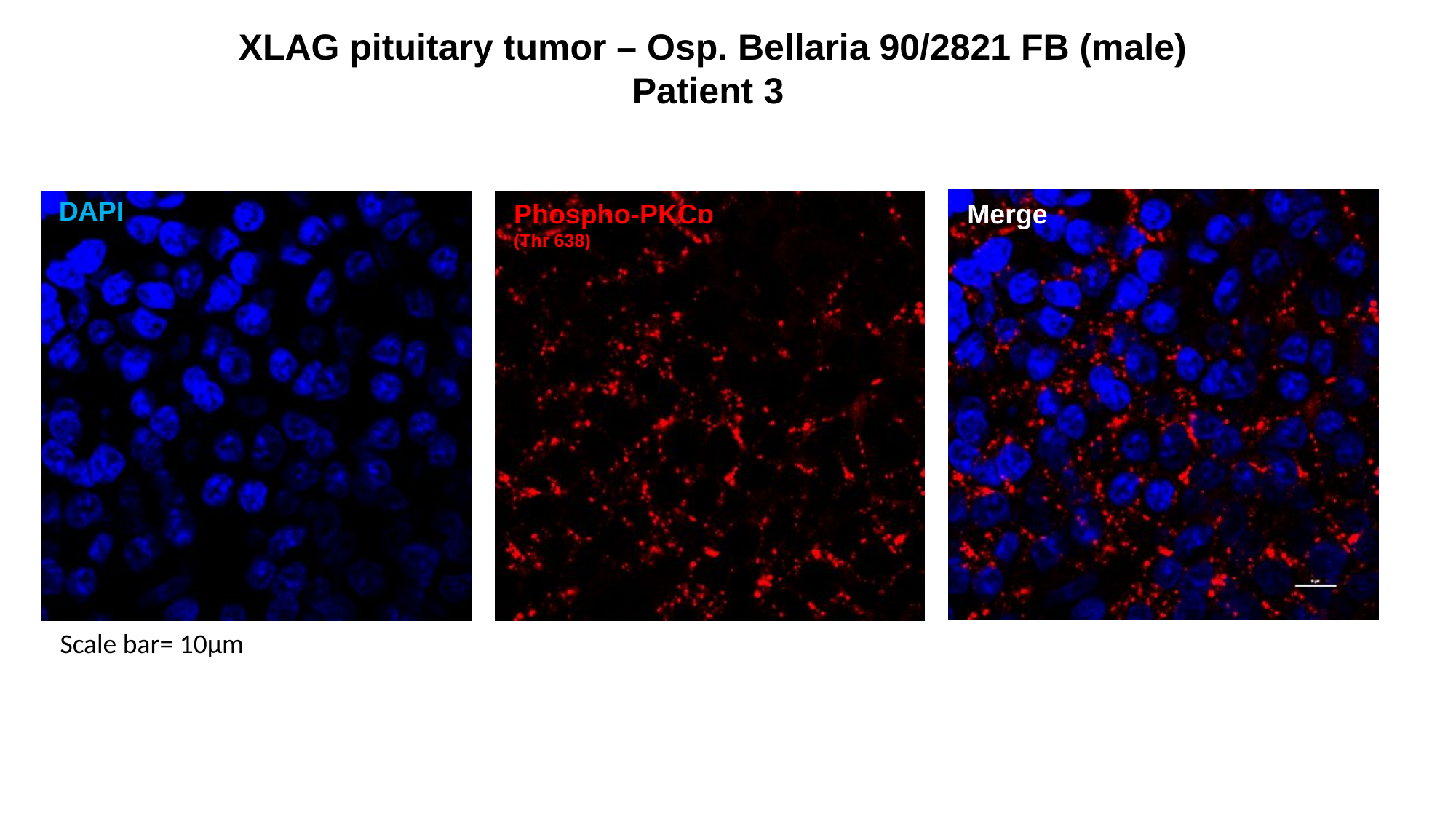

XLAG pituitary tumor – Osp. Bellaria 90/2821 FB (male)
Patient 3
DAPI
Phospho-PKCɒ
(Thr 638)
Merge
Scale bar= 10µm
