# Supplementary material for: GPR101 drives growth hormone hypersecretion and gigantism in mice via constitutive activation of Gs and Gq/11
Source: Nat Commun. 2020 Sep 21;11:4752. doi: 10.1038/s41467-020-18500-x (PMC7506554; doi:10.1038/s41467-020-18500-x)
Supplement: Supplementary file 4 — Source Data [file 41467_2020_18500_MOESM4_ESM.zip › Source Data/Source data - Figure 3 - Panel D.pptx]

## Slide 1
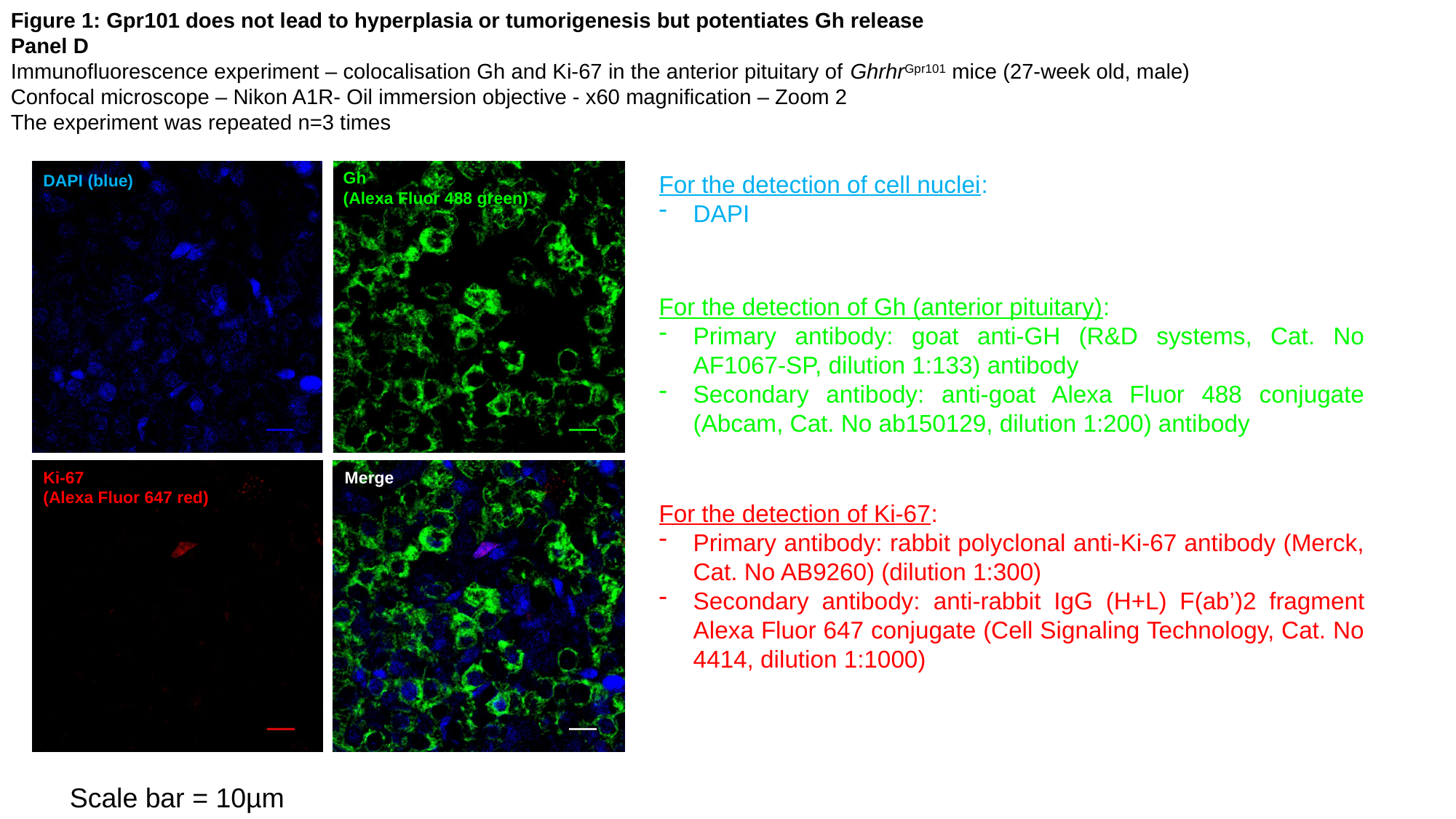

Figure 1: Gpr101 does not lead to hyperplasia or tumorigenesis but potentiates Gh release
Panel D
Immunofluorescence experiment – colocalisation Gh and Ki-67 in the anterior pituitary of GhrhrGpr101 mice (27-week old, male)
Confocal microscope – Nikon A1R- Oil immersion objective - x60 magnification – Zoom 2
The experiment was repeated n=3 times
Gh
(Alexa Fluor 488 green)
For the detection of cell nuclei:
DAPI
DAPI (blue)
For the detection of Gh (anterior pituitary):
Primary antibody: goat anti-GH (R&D systems, Cat. No AF1067-SP, dilution 1:133) antibody
Secondary antibody: anti-goat Alexa Fluor 488 conjugate (Abcam, Cat. No ab150129, dilution 1:200) antibody
Ki-67
(Alexa Fluor 647 red)
Merge
For the detection of Ki-67:
Primary antibody: rabbit polyclonal anti-Ki-67 antibody (Merck, Cat. No AB9260) (dilution 1:300)
Secondary antibody: anti-rabbit IgG (H+L) F(ab’)2 fragment Alexa Fluor 647 conjugate (Cell Signaling Technology, Cat. No 4414, dilution 1:1000)
Scale bar = 10µm
