# Supplementary material for: GPR101 drives growth hormone hypersecretion and gigantism in mice via constitutive activation of Gs and Gq/11
Source: Nat Commun. 2020 Sep 21;11:4752. doi: 10.1038/s41467-020-18500-x (PMC7506554; doi:10.1038/s41467-020-18500-x)
Supplement: Supplementary file 4 — Source Data [file 41467_2020_18500_MOESM4_ESM.zip › Source Data/Source data - Figure 2 - Panel E.pptx]

## Slide 1
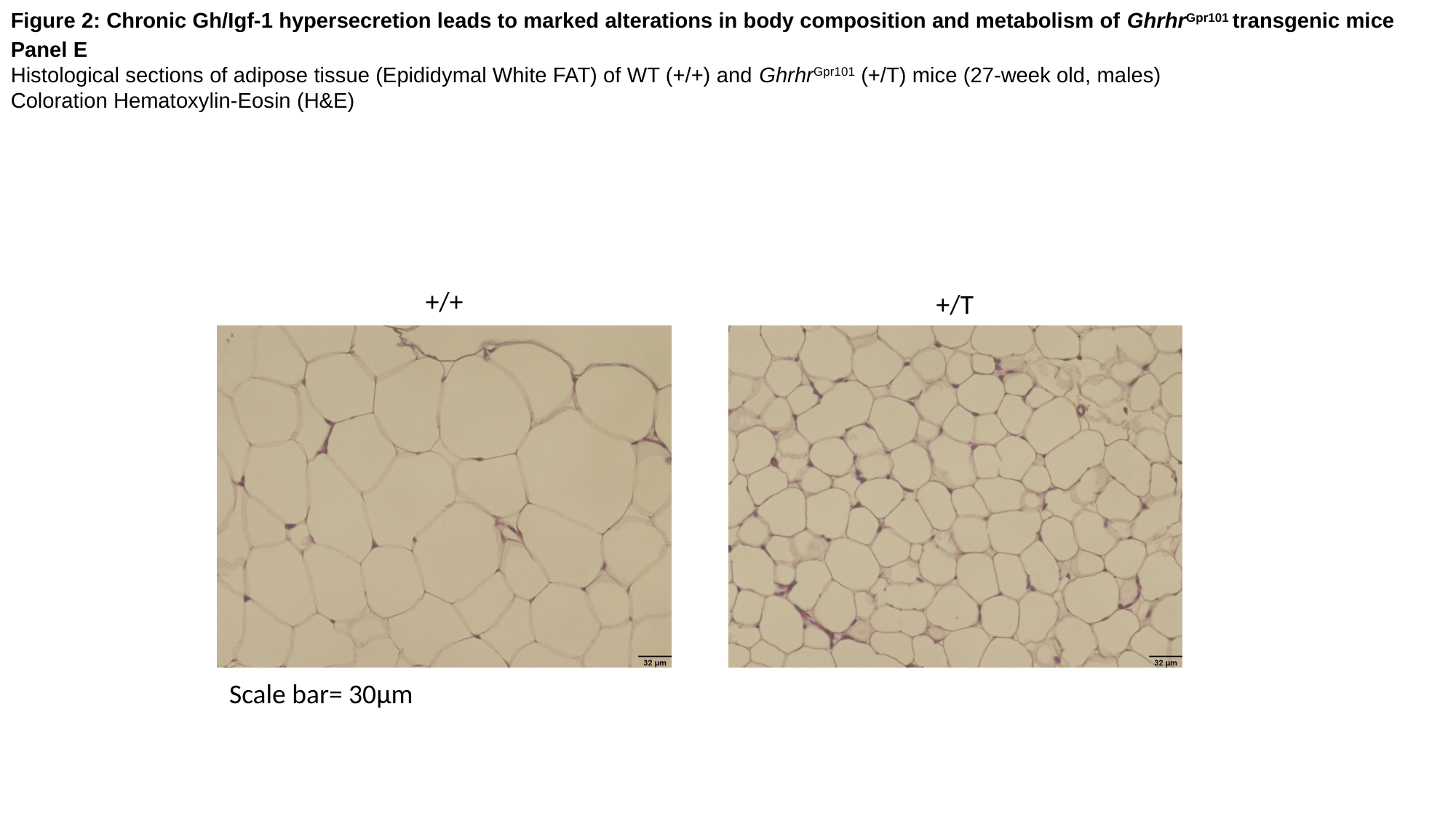

Figure 2: Chronic Gh/Igf-1 hypersecretion leads to marked alterations in body composition and metabolism of GhrhrGpr101 transgenic mice
Panel E
Histological sections of adipose tissue (Epididymal White FAT) of WT (+/+) and GhrhrGpr101 (+/T) mice (27-week old, males)
Coloration Hematoxylin-Eosin (H&E)
+/+
+/T
Scale bar= 30µm
